# Itaconate is a metabolic regulator of bone formation in homeostasis and arthritis

**DOI:** 10.1136/ard-2023-224898

**Published:** 2024-07-10

**Authors:** Markus Kieler, Leona Sophia Prammer, Gerwin Heller, Melanie Hofmann, Simon Sperger, Dominik Hanetseder, Birgit Niederreiter, Andrea Komljenovic, Kristaps Klavins, Thomas Köcher, Julia Stefanie Brunner, Irena Stanic, Laura Oberbichler, Ana Korosec, Andrea Vogel, Martina Kerndl, Dominika Hromadová, Laszlo Musiejovsky, Alexander Hajto, Anja Dobrijevic, Tina Piwonka, Arvand Haschemi, Anne Miller, Philippe Georgel, Darja Marolt Presen, Johannes Grillari, Silvia Hayer, Jean-Philippe Auger, Gerhard Krönke, Omar Sharif, Daniel Aletaha, Gernot Schabbauer, Stephan Blüml

**Affiliations:** 1 Institute for Vascular Biology, Centre for Physiology and Pharmacology, Medical University of Vienna, Wien, Vienna, Austria; 2 Christian Doppler Laboratory for Arginine Metabolism in Rheumatoid Arthritis and Multiple Sclerosis, Vienna, Austria; 3 Department of Rheumatology, Medical University of Vienna, Wien, Vienna, Austria; 4 Department of Medicine I, Division of Oncology, Medical University of Vienna, Vienna, Austria; 5 Ludwig Boltzmann Institute for Experimental and Clinical Traumatology, Wien, Vienna, Austria; 6 Austrian Cluster for Tissue Regeneration, Vienna, Austria; 7 Christian Doppler Laboratory for Immunometabolism and Systems Biology of Obesity-Related Diseases (InSpiReD), Vienna, Austria; 8 Institute of General Chemical Engineering, Riga Technical University, Riga, Latvia; 9 Vienna BioCenter Core Facilities, Campus-Vienna-BioCenter 1, Vienna, Austria; 10 Cell Biology Program, Memorial Sloan Kettering Cancer Center, New York, New York, USA; 11 Institute of Experimental Immunology, University of Zurich, Zurich, Switzerland; 12 Department of Laboratory Medicine, Medical University of Vienna, Wien, Austria; 13 Center for Pathobiochemistry and Genetics, Medical University of Vienna, Vienna, Austria; 14 INSERM UMR_S 1109, Fédération de Médecine Translationnelle (FMTS), Université de Strasbourg, Centre de Recherche en Immunologie et Hématologie, 1 Place de l’Hôpital, Strasbourg Cedex, France; 15 Institute of Molecular Biotechnology, University of Natural Resources and Life Sciences, Vienna, Austria; 16 Department of Internal Medicine 3 - Rheumatology and Immunology, Friedrich-Alexander University Erlangen-Nürnberg and Universitätsklinikum Erlangen, Erlangen, Germany

**Keywords:** bone density, arthritis, experimental, spondylitis, ankylosing

## Abstract

**Objectives:**

Bone remodelling is a highly dynamic process dependent on the precise coordination of osteoblasts and haematopoietic-cell derived osteoclasts. Changes in core metabolic pathways during osteoclastogenesis, however, are largely unexplored and it is unknown whether and how these processes are involved in bone homeostasis.

**Methods:**

We metabolically and transcriptionally profiled cells during osteoclast and osteoblast generation. Individual gene expression was characterised by quantitative PCR and western blot. Osteoblast function was assessed by Alizarin red staining. immunoresponsive gene 1 (*Irg1*)*-*deficient mice were used in various inflammatory or non-inflammatory models of bone loss. Tissue gene expression was analysed by RNA in situ hybridisation.

**Results:**

We show that during differentiation preosteoclasts rearrange their tricarboxylic acid cycle, a process crucially depending on both glucose and glutamine. This rearrangement is characterised by the induction of *Irg1* and production of itaconate, which accumulates intracellularly and extracellularly. While the IRG1–itaconate axis is dispensable for osteoclast generation in vitro and in vivo, we demonstrate that itaconate stimulates osteoblasts by accelerating osteogenic differentiation in both human and murine cells. This enhanced osteogenic differentiation is accompanied by reduced proliferation and altered metabolism. Additionally, supplementation of itaconate increases bone formation by boosting osteoblast activity in mice. Conversely, *Irg1-*deficient mice exhibit decreased bone mass and have reduced osteoproliferative lesions in experimental arthritis.

**Conclusion:**

In summary, we identify itaconate, generated as a result of the metabolic rewiring during osteoclast differentiation, as a previously unrecognised regulator of osteoblasts.

**WHAT IS ALREADY KNOWN ON THIS TOPIC**

- Itaconate is a metabolite produced by myeloid cells with potent immunoregulatory properties.

**WHAT THIS STUDY ADDS**

- Our study demonstrates that itaconate is induced during osteoclast generation and identifies a role of itaconate in the regulation of bone biology by stimulating osteogenic differentiation.

**HOW THIS STUDY MIGHT AFFECT RESEARCH, PRACTICE OR POLICY**

- Our study opens avenues for using itaconate or derivatives for the treatment of diseases affecting bones and joints.

## Introduction

 Osteoclasts are multinucleated cells of myeloid origin with an essential role in skeletal homeostasis due to their unique ability to resorb bone,^1^ which act in conjunction with osteoanabolic cells such as osteoblasts and osteocytes. Cellular polarisation or differentiation of immune cells is accompanied by metabolic adaptations which reflect changed energetic requirements for novel cellular functions.^2^ Previous work on the metabolic regulation of osteoclastogenesis has focused on the terminal differentiation process, which is characterised by an upregulation of mitochondrial biogenesis and subsequent increases in mitochondrial respiration.^3^,^7^ This shift towards oxidative metabolism in the late phase of osteoclastogenesis supports epigenetic remodelling important for repression of antiosteoclastogenic genes.^6^ However, changes in cellular metabolism during early osteoclastogenesis and whether they are involved in maintaining bone homeostasis are poorly understood.^8^,^11^

## Results

**Figure 1:**
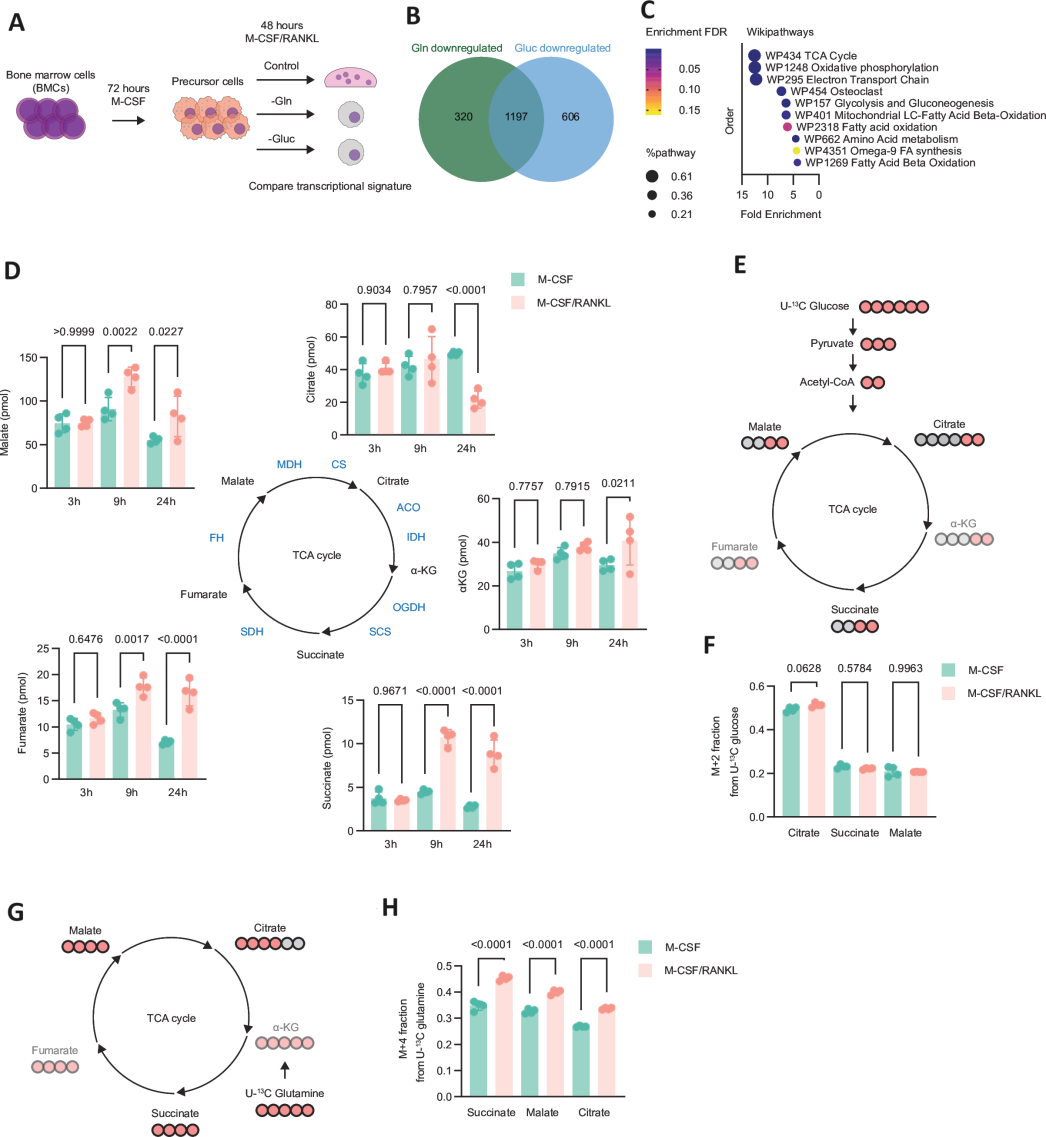
RANKL changes cellular metabolism of differentiating osteoclasts. (A) Experimental setup of RNA-seq workflow. BMCs were stimulated with M-CSF for 72 hours and then replated with M-CSF/RANKL for 48 hours in medium lacking either glutamine or glucose. (B) Venn diagram depicting the overlap between downregulated genes (FC<−1.5 and FDR<0.05) in cells stimulated as shown in (A) compared with cells cultivated in the control medium (n=3). (C) Top 10 enriched pathways with >5 genes/pathway from 1197 overlapping genes from (B) assessed by GO enrichment analysis. Colour denotes FDR and size of the shape denotes affected genes in relation to all genes from a particular pathway. (D) Absolute abundance of TCA cycle intermediates in cells stimulated with either M-CSF or M-CSF/RANKL for 3 hours, 9 hours and 24 hours (n=4). (E) Schematic depicting the fate of U-^13^C labelled atoms derived from U-^13^C glucose. Light red circles indicate labelled C-atoms. (F) M+2 fraction of the total pool for analysed TCA cycle intermediates in cells cultivated in medium supplemented with U-^13^C glucose and stimulated with either M-CSF or M-CSF/RANKL for 6 hours (n=4). (G) Schematic depicting the fate of U-^13^C labelled atoms derived from U-^13^C glutamine. Light red circles indicate labelled C-atoms. (H) M+4 fraction of the total pool for analysed TCA cycle intermediates in cells cultivated in medium supplemented with U-^13^C glutamine and stimulated with either M-CSF or M-CSF/RANKL for 6 hours (n=4). The data are represented as means±SDs. One-way ANOVA with Šidàk’s correction (D, F, H). BMCs, bone marrow-derived cells; RANKL, receptor activator of nuclear factor κB ligand; TCA, tricarboxylic acid.

To better understand metabolic changes during osteoclast differentiation, we determined the functional importance of the two core nutrients glucose and glutamine, which both have been suggested to play a role in osteoclastogenesis.^12 13^ Thus, we cultivated bone marrow-derived cells (BMCs) in medium deficient in either glucose or glutamine and stimulated them with the primary cytokine mediating osteoclast differentiation receptor activator of nuclear factor κB ligand (RANKL). In line with previous results, both nutrients were required for osteoclast differentiation (online supplemental figure S1a, b).^12 13^ To comprehensively assess glucose or glutamine osteoclastogenesis-associated transcriptional programmes, we performed RNA sequencing of BMCs stimulated with RANKL in the presence or absence of glutamine or glucose for 48 hours (figure 1A). Analysis of the differentially downregulated genes revealed a substantial overlap between glucose and glutamine deficiency with enrichment analysis indicating these shared genes impinged significantly on tricarboxylic acid (TCA) cycle and oxidative phosphorylation-associated pathways (figure 1B, C and online supplemental table 1).

Therefore, we analysed the absolute levels of TCA cycle metabolites during early (24 hours) RANKL-induced osteoclastogenesis and could observe profound changes in the pools of TCA cycle metabolites between 3 and 9 hours (figure 1D). Specifically, compared with levels in unstimulated cells succinate, fumarate and malate markedly increased and remained elevated up to 24 hours, whereas citrate levels decreased 24 hours after RANKL stimulation. Using stable isotope tracing, we determined TCA cycle fuels during osteoclast differentiation. Therefore, we cultivated cells in medium containing both U-^13^C glucose and U-^13^C glutamine, stimulated them with RANKL and found that all major TCA intermediates such as citrate, succinate and malate were labelled (online supplemental figure S1c, d). In the next step, we aimed to determine the individual contributions of these TCA cycle fuels therein (figure 1E, G). We assessed the relative pools of TCA cycle intermediates labelled from either U-^13^C glucose or U-^13^C glutamine and did not detect changes in the M+2 (containing two ^13^C isotopes) fractions from ^13^C glucose 6 hours post RANKL stimulation (figure 1E, F). In contrast, RANKL stimulation led to a marked increase in the M+4 (containing four ^13^C isotopes) fractions from U-^13^C glutamine of analysed TCA cycle intermediates, which suggested a shift towards glutamine-derived carbon fluxes to replenish TCA cycle intermediates in differentiating osteoclasts (figure 1G, H). In line with glutamine feeding into the TCA cycle via conversion to α-ketoglutarate (α-KG), supplementation with a cell permeable derivative of α-KG rescued defective osteoclast differentiation in glutamine-deficient medium (online supplemental figure S1e, f). The increase in glutamine-derived TCA cycle intermediates was transient and returned to baseline levels after 48 hours (online supplemental figure S1g). Isotopologue distributions showed decreased labelling of U-^13^C glutamine-derived citrate and succinate and increased labelling of succinate derived from U-^13^C glucose 48 hours after RANKL stimulation compared with 24 hours, suggesting increased glucose and reduced glutamine TCA cycle anaplerosis (online supplemental figure S1h, i). Moreover, RANKL induced increase in succinate levels was also transient (online supplemental figure S1j). Taken together, we found that osteoclast differentiation induces early and profound changes in the TCA cycle of preosteoclasts, which is most clearly characterised by succinate accumulation and a boost in glutamine-fueled anaplerosis. These observations are reminiscent of classically activated macrophages, where a so-called broken TCA cycle is linked to the expression of mitochondria-associated immunoresponsive gene 1 (*Irg1*).^14^,^16^

**Figure 2:**
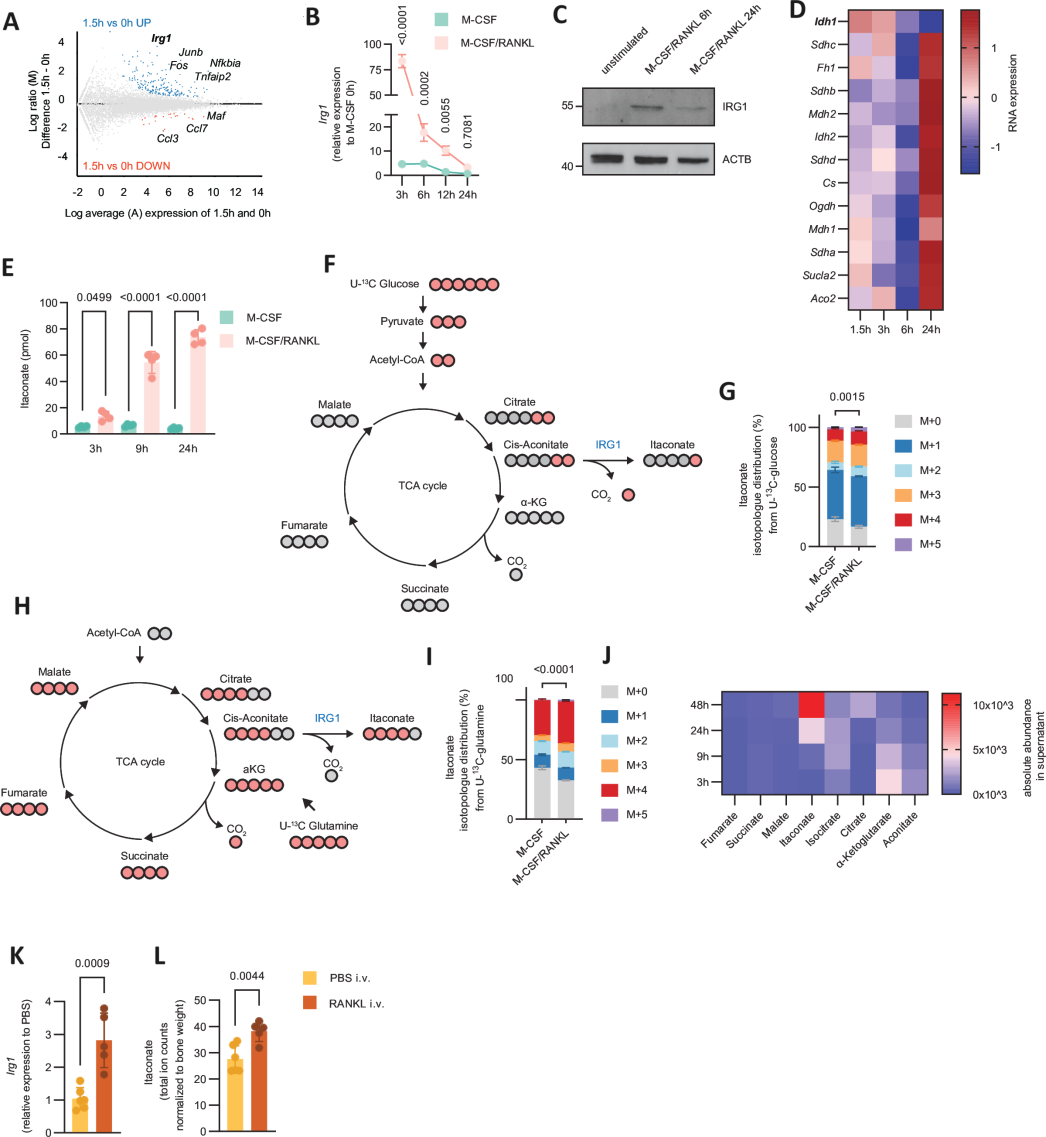
RANKL induces *Irg1* expression and itaconate production in differentiating osteoclasts. (A) MA plot for differentially expressed genes in BMCs stimulated with M-CSF/RANKL for 1.5 hours versus unstimulated cells (M-CSF 0 hour) (shrunk log2 FC>0.585, FDR<0.05) (n=3). Each dot represents a transcript. Blue, upregulated; red, downregulated; grey, no difference. (B) Relative mRNA expression of *Irg1* compared with RANKL unstimulated BMCs (M-CSF 0 hour) during a time course of RANKL stimulation (3 hours, 6 hours, 12 hours, 24 hours) using real-time PCR (n=4). (C) Immunoblot of IRG1 from protein lysates of RANKL unstimulated (M-CSF 0 hour), 6 hours and 24 hours M-CSF/RANKL stimulated BMCs. (D) Heatmap of genes encoding for TCA cycle biosynthetic enzymes during a time course of 1.5 hours, 3 hours, 6 hours and 24 hours post RANKL stimulation of BMCs determined by RNA-seq (n=3). Mean row centred/scaled expression values are shown. (E) Absolute levels of itaconate from cell extracts of M-CSF or M-CSF/RANKL stimulated BMCs over a time course of 3 hours, 9 hours and 24 hours (n=4). (F) Schematic depicting the fate of U-^13^C labelled atoms for itaconate derived from U-^13^C glucose. Light red circles indicate labelled C-atoms. (G) Relative isotopologue distribution of itaconate from cell extracts of M-CSF or M-CSF/RANKL stimulated BMCs for 6 hours in medium supplemented with U-^13^C glucose (n=4). (H) Schematic of the fate of the U-^13^C labelled atoms for itaconate derived from U-^13^C glutamine (n=4). Light red circles indicate labelled C-atoms. (I) Relative isotopologue distribution of itaconate from cell extracts of M-CSF or M-CSF/RANKL stimulated BMCs for 6 hours in medium supplemented with U-^13^C glutamine (n=4). (J) Heatmap depicting the absolute abundance of TCA cycle intermediates in the supernatant from BMCs stimulated with M-CSF/RANKL at 3 hours, 9 hours, 24 hours and 48 hours. (K) Relative mRNA expression of *Irg1* in tibial bone isolated from mice 3–6 hours after intravenous injection with PBS or RANKL using real-time PCR (n=5–6). (L) Itaconate levels of tibial bone normalised to bone weight isolated from mice as shown in (K) (n=5–6). The data are represented as means±SDs. Two-way ANOVA with Šidàk’s correction (B), one-way ANOVA with Šidàk’s correction (E) and unpaired t-test of M+0 values (G, I, K, L). BMCs, bone marrow-derived cells; *Irg1*, immunoresponsive gene 1; PBS, phosphate buffered saline; RANKL, receptor activator of nuclear factor κB ligand; TCA, tricarboxylic acid.

We therefore tested whether *Irg1* is expressed post RANKL stimulation and found that *Irg1* was among the most highly RANKL-induced genes with upregulation occurring as early as 90 min post RANKL stimulation in primary BMCs and the myeloid precursor cell line HoxB8 (figure 2A, online supplemental figure 2A and online supplemental table 2).^17^ Sorted human CD14^+^ monocytes also upregulated *IRG1* in a similar manner when stimulated with RANKL (online supplemental figure S2b). *Irg1* expression peaked at 3 hours and subsequently returned to baseline levels after 24 hours, which was confirmed by immunoblotting (figure 2B, C and online supplemental figure S2c). Concomitantly, and in line with previous observations in classically activated macrophages, isocitrate dehydrogenase 1 (*Idh1*) was downregulated (figure 2D).^14^ Robust IRG1 expression was associated with an increase in itaconate levels which started at 3 hours, accumulated until 24 hours post RANKL stimulation and decreased thereafter (figure 2E and online supplemental figure S2d). RANKL stimulation increased the labelled fraction of itaconate derived from both U-^13^C glucose and U-^13^C glutamine, while the unlabelled fraction in cells cultured with U-^13^C glucose was substantially smaller compared with that in cells cultured with U-^13^C glutamine (figure 2F–I). These data show that production of itaconate is a distinct feature associated with TCA cycle rewiring during osteoclast differentiation and that glucose-derived and to a lesser extent also glutamine-derived carbon fluxes via the TCA cycle are rerouted towards itaconate.

We further asked whether RANKL stimulated BMCs, akin to macrophages on encountering proinflammatory stimuli, would secrete itaconate.^16 18^ Indeed, we detected itaconate accumulation in the supernatant of differentiating osteoclasts which resembled the dynamics of *Irg1* expression and the increase in intracellular itaconate pools (online supplemental figure S2e). Itaconate was also the most abundant TCA cycle metabolite in the supernatant of differentiating osteoclasts over a time course of RANKL stimulation (figure 2J). We further traced U-^13^C glucose into secreted TCA cycle metabolites and found that the labelled fraction of succinate and malate in the supernatant was two to four times lower than that of itaconate and that the labelling pattern of extracellular itaconate resembled that of the intracellular pool (online supplemental figure S2f, g). Of note, comparing *Irg1* expression among different organs, we found that bone had higher expression than lung, kidney or liver (online supplemental figure S2h). Consistent with its effect on osteoclast precursors in vitro, intravenous injection of RANKL into mice resulted in increased *Irg1* expression and itaconate levels in the bone while having negligible effects on itaconate serum levels (figure 2K, L and online supplemental figure S2i, j). Together, these data show that itaconate is an abundantly secreted metabolite by differentiating osteoclasts on RANKL stimulation in vitro and in vivo.

**Figure 3:**
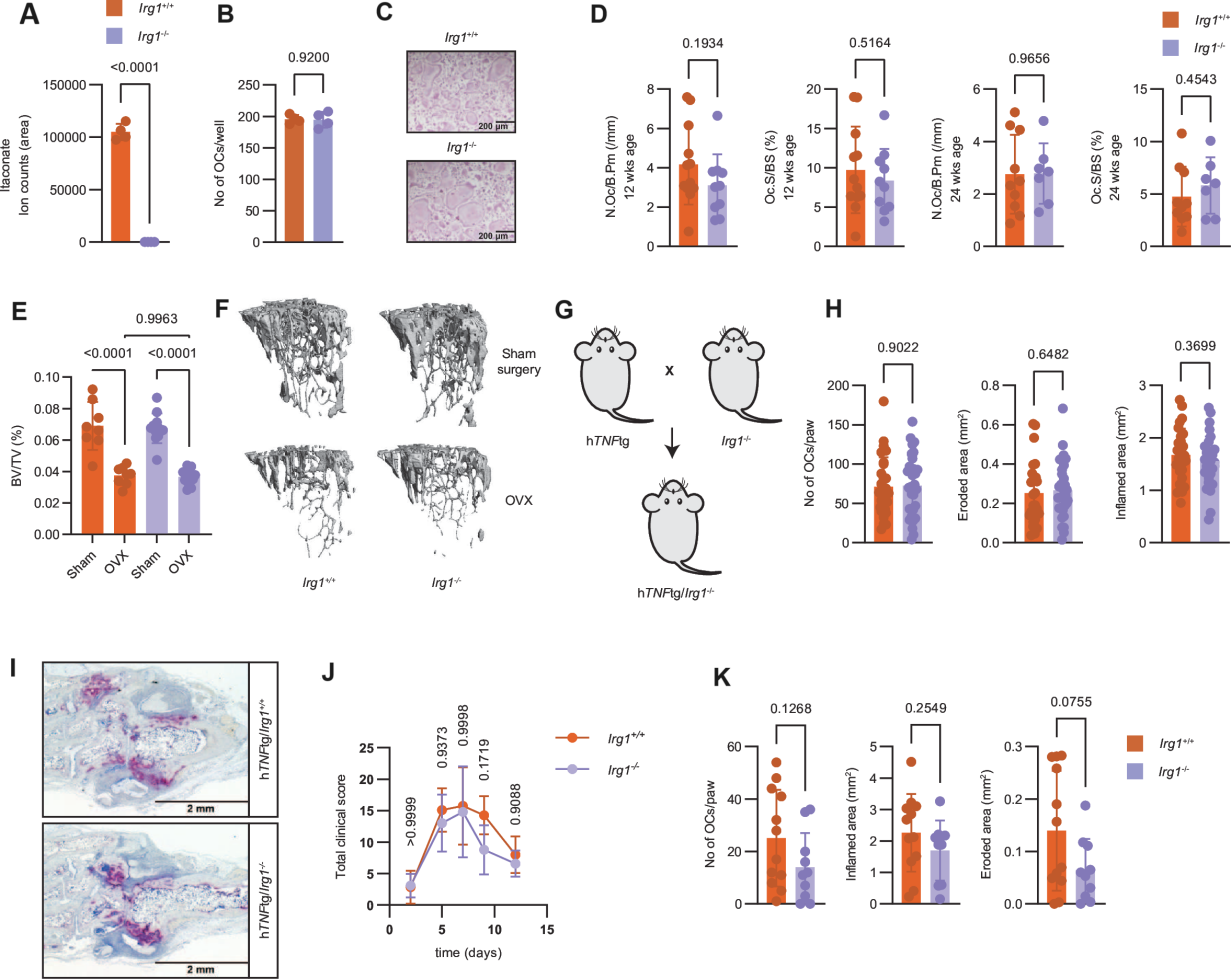
*Irg1* is dispensable for osteoclastogenesis in vitro and in vivo. (A) Intracellular levels of itaconate from cell extracts of *Irg1^+/+^* or *Irg1^−/−^* BMCs stimulated with M-CSF/RANKL for 6 hours (n=4). (B) Number of osteoclasts per well from *Irg1^+/+^* or *Irg1^−/−^* BMCs stimulated with M-CSF/RANKL (n=4). (C) Representative images of TRAP-stained *Irg1^+/+^* or *Irg1^−/−^* BMCs stimulated with M-CSF/RANKL for 96 hours. Scale bar represents 200 µm. (D) Number of osteoclasts per bone perimeter (N.Oc/B.Pm) and osteoclast surface per bone surface (Oc.S/BS) from 12-weeks-old and 24-weeks-old *Irg1^+/+^* or *Irg1^−/−^* mice (n=7–12). (E) Trabecular bone volume per tissue volume (BV/TV (%)) from *Irg1^+/+^* or *Irg1^−/−^* mice after ovariectomy (OVX) or sham surgery (n=8). (F) Representative 3D reconstructions of tibial bones from (E). (G) Scheme for the generation of h*TNF*tg/*Irg1^+/+^* or h*TNF*tg*/Irg1^−/−^* mice. (H) Number of osteoclasts per section of hind paws (No of OCs/paw), eroded area (mm^2^) and inflamed area (mm^2^) from 8-weeks-old h*TNF*tg/*Irg1^+/+^* or h*TNF*tg*/Irg1^−/−^* (n=14–16). (I) Representative images of TRAP-stained histological slides from hind paws of h*TNF*tg/*Irg1^+/+^* or h*TNF*tg*/Irg1^−/−^* mice. Scale bar represents 2 mm. (J) Total clinical score of *Irg1^+/+^* or *Irg1^−/−^* mice injected with K/BxN serum at indicated time points after injections (n=5–6). (K) Number of osteoclasts per section of hind paws (No of OCs/paw), inflamed area (mm^2^) and eroded area (mm^2^) from mice in (J). The data are represented as means±SDs. Unpaired t-test (A, B, D, H, K), one1-way ANOVA with Šidàk’s correction (E) and two-way ANOVA with Šidàk’s correction (J). ANOVA, analysis of variance; BMCs, bone marrow-derived cells; *Irg1*, immunoresponsive gene 1; RANKL, receptor activator of nuclear factor κB ligand.

To understand the functional relevance of *Irg1* induction and itaconate production for osteoclastogenesis, we tested whether *Irg1* deficiency affects osteoclast generation. Using LC-MS/MS, we first confirmed that loss of IRG1 abolishes the conversion of cis-aconitate to itaconate in differentiating osteoclasts, as itaconate was undetectable in *Irg1^−/−^* cells after RANKL stimulation (figure 3A and online supplemental figure S3a). However, we did not detect differences in the number of tartrate-resistant acid phosphatase (TRAP)^+^ multinuclear cells and RNA expression of osteoclast-related genes between the genotypes, which shows that *Irg1* is dispensable for osteoclast differentiation from BMCs in vitro (figure 3B, C and online supplemental S3b). To determine the contribution of *Irg1* to osteoclastogenesis in vivo, we assessed osteoclast-related parameters such as osteoclast number per bone perimeter (N.Oc/BPm) or osteoclast surface per bone surface (Oc.S/BS) in tibial bones of *Irg1*^+/+^ and *Irg1*^−/−^ mice by histomorphometry. Osteoclast-associated parameters were not different between genotypes at 12 and 24 weeks of age (figure 3D). In addition, serum levels of C telopeptide of type I collagen (CTX-1) were also unchanged (online supplemental figure S3c). To investigate, whether *Irg1* plays a role in osteoclast-mediated disease models, we ovariectomised *Irg1*^+/+^ and *Irg1*^−/−^ mice to analyse bone loss induced by oestrogen deprivation.^19^ Compared with sham surgery, both *Irg1*^+/+^ and *Irg1*^−/−^ mice showed significant loss of bone mass after ovariectomy. However, bone loss was not different between genotypes (figure 3E, F). To test whether *Irg1* is involved in osteoclastogenesis induced during joint inflammation, we used h*TNF*tg mice in which overexpression of human *TNF* causes a chronic inflammatory arthritis leading to osteoclast formation at the site of joint inflammation,^20^ and intercrossed them with *Irg1^−/−^* mice (figure 3G). Analysis of histological parameters of arthritis including the number of osteoclasts or the extent of osteoclast-mediated erosive joint destruction revealed no differences between h*TNF*tg/*Irg1^+/+^* and h*TNF*tg/*Irg1^−/−^* animals (figure 3H). In addition, there was no difference with regard to overall joint inflammation suggesting that *Irg1* does not affect the course of TNF-driven arthritis (figure 3H; representative TRAP staining figure 3I). Assessing a potential role of *Irg1* in another model of arthritis, the K/BxN serum transfer arthritis model,^21^ again showed no clear differences between *Irg1*^+/+^ and *Irg1*^−/−^ mice in both the clinical course of arthritis and osteoclast-related parameters in histological sections (figure 3J, K and online supplemental figure S3e, f). Thus, we conclude that *Irg1* is dispensable for osteoclast differentiation also in vivo.

**Figure 4:**
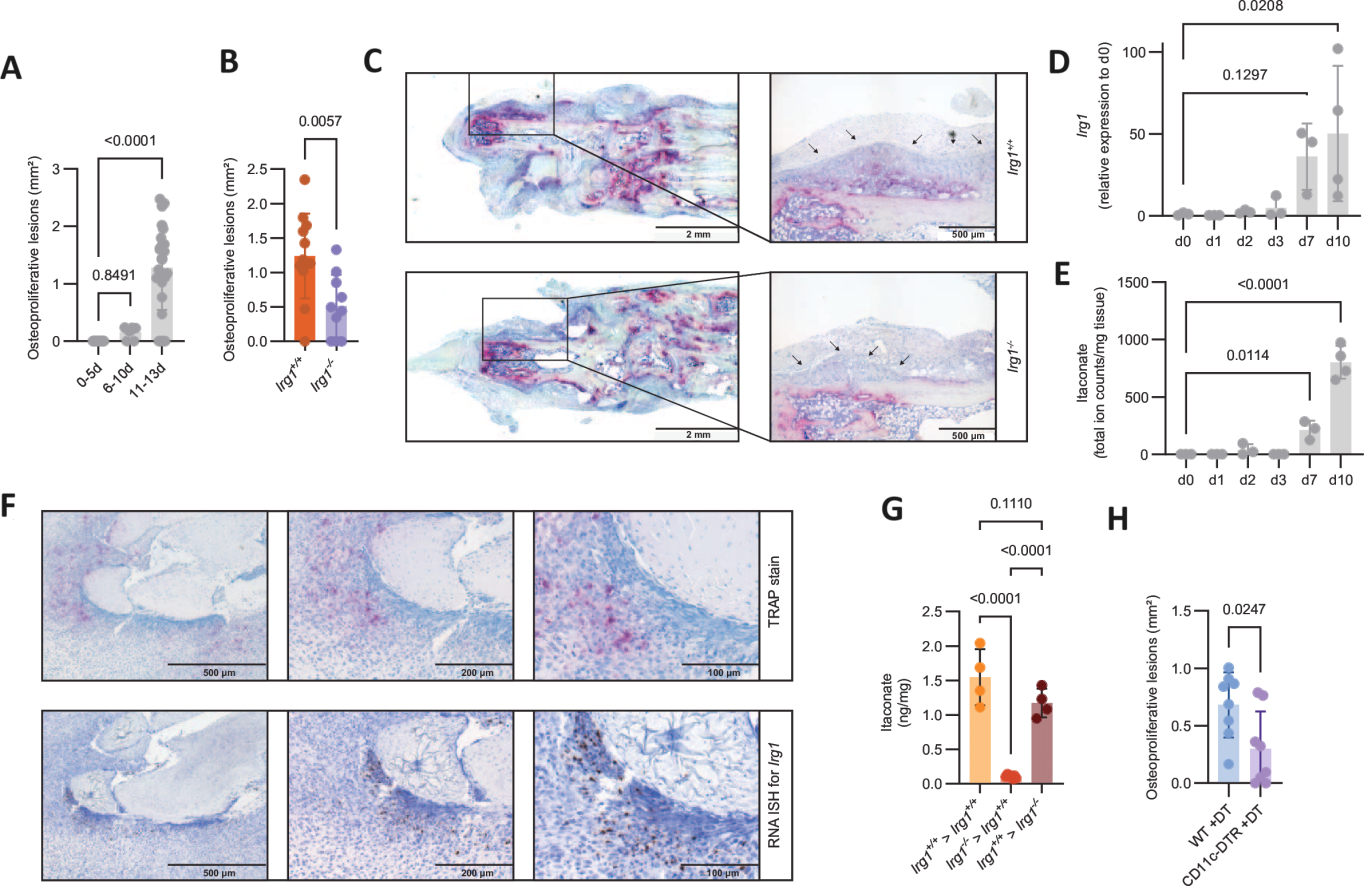
*Irg1* expression and itaconate influence new bone formation in arthritis. (A) Area of osteoproliferative lesions (in mm^2^) in hind paws of mice injected with K/BxN serum at indicated time points after injection (n=7–26). (B) Area of osteoproliferative lesions (in mm^2^) in hind paws from *Irg1^+/+^* or *Irg1^−/−^* mice injected with K/BxN serum (n=10–12). (C) Representative images of TRAP-stained histological slides from hind paws of mice shown in (B). Black arrows indicate the boundaries of the osteoproliferative lesions. Scale bars represent 2 mm and 500 µm. (D, E) Relative mRNA expression of *Irg1* (D) and itaconate levels (E) in paws isolated from mice injected with serum from K/BxN mice harvested at indicated time points (n=3–4). (F) Representative images of histological slides of an inflamed paw (K/BxN serum transfer arthritis model) stained for TRAP (purple staining indicates TRAP^+^ cells) or *Irg1* expression as detected with RNA ISH (brown staining indicates *Irg1* expressing cells). Scale bars represent 500 µm, 200 µm and 100 µm. (G) Itaconate levels in paws from bone marrow transplanted mice injected with serum from K/BxN mice on day nine post injection. Lethally irradiated *Irg1^+/+^* and *Irg1^−/−^* mice were transplanted with *Irg1^+/+^* and *Irg1^−/−^* bone marrow (*Irg1^+/+^* bone marrow to *Irg1^+/+^* mice (*Irg1^+/+^>Irg1^+/+^*), *Irg1^−/−^* bone marrow to *Irg1^+/+^* mice (*Irg1^−/−^>Irg1^+/+^*), *Irg1^+/+^* bone marrow to *Irg1^−/−^* mice (*Irg1^+/+^>Irg1^−/−^*) (n=4–7). (H) Area of osteoproliferative lesions (in mm^2^) of hind paws from wildtype or CD11c-DTR mice injected with serum from K/BxN mice and treated with diphtheria toxin (n=8). The data are represented as means±SDs. One-way ANOVA with Šidàk’s correction (A, D, E, G) and unpaired t-test (B, H). ANOVA, analysis of variance; *Irg1*, immunoresponsive gene 1; ISH, *in situ* hybridisation.

In the K/BxN serum transfer arthritis model, the development of erosive arthritis is accompanied by significant osteoblast-mediated new bone formation, reminiscent of osteoproliferative lesions found in patients with spondyloarthritis,^22 23^ which formed in the later stages of this disease model (figure 4A). Intriguingly, we found markedly reduced osteoproliferation in arthritic paws from *Irg1*^−/−^ compared with *Irg1*^+/+^ mice, suggesting a functional role of *Irg1* and itaconate in the formation of these lesions (figure 4B, C). In line with the temporal appearance of the osteoproliferative lesions, we also detected notably increased *Irg1* expression and itaconate levels in the inflamed paws during the later course of arthritis (figure 4D, E), which was also paralleled by increased expression of the myeloid marker genes *Itgam* and *Emr1* (online supplemental figure S4a). Using RNA in situ hybridisation we further observed that *Irg1* expressing cells were not distributed randomly among inflamed areas but preferably localised together with clusters of TRAP^+^ cells around the tendons and their insertion into the bone (figure 4F and online supplemental figure S4b), which is a site of predilection for osteoproliferative lesions.^24^ To further delineate resident versus invading myeloid cells as the main producing myeloid cell population, we transplanted bone marrow from *Irg1^+/+^* into *Irg1^−/−^* recipients or vice versa and measured paw swelling and itaconate in the inflamed paws after induction of K/BxN serum transfer arthritis. While paw swelling was similar in all groups (online supplemental figure S4c), engraftment of *Irg1^−/−^* bone marrow cells in *Irg1^+/+^*-recipient mice completely abolished itaconate accumulation in the arthritic paws, while in the opposite scenario itaconate levels were fully normalised (figure 4G). Taken together, these results show that itaconate is predominantly produced by invading myeloid populations, which are attracted to the inflamed tissue in the later phases of arthritis leading to accumulation of this metabolite at predilection sites of osteoproliferative lesions. Further supporting a role of osteoclasts as contributors to osteoproliferation in the K/BxN serum transfer arthritis model, we used a mouse model, in which a genetic construct encoding the diphtheria toxin receptor is fused to the CD11c promoter region (CD11c-DTR),^25^ which we recently showed to target osteoclasts and their precursors.^26^ In line with our previous observations, administration of diphtheria toxin during K/BxN serum transfer arthritis did not reduce the inflamed area in the paws of CD11c-DTR mice compared with controls (online supplemental figure S4d), but reduced the number of osteoclasts (online supplemental figure S4f). Of note, depletion of CD11c^+^ cells significantly reduced osteoproliferative lesions (figure 4H), demonstrating that osteoclasts and their precursors contribute to new bone formation in this model. Furthermore, administration of diphtheria toxin leads to depletion of CD11c^+^ cells including osteoclasts and their precursors in tibial bones (online supplemental figure S4g), which is followed by a subsequent repopulation with osteoclasts starting 3 days after the last diphtheria toxin administration.^26^ Using this model, we detected increased *Irg1* expression in the tibiae of mice during this repopulation phase, again underlining that this gene is also linked to osteoclast differentiation in vivo (online supplemental figure S4h).

**Figure 5:**
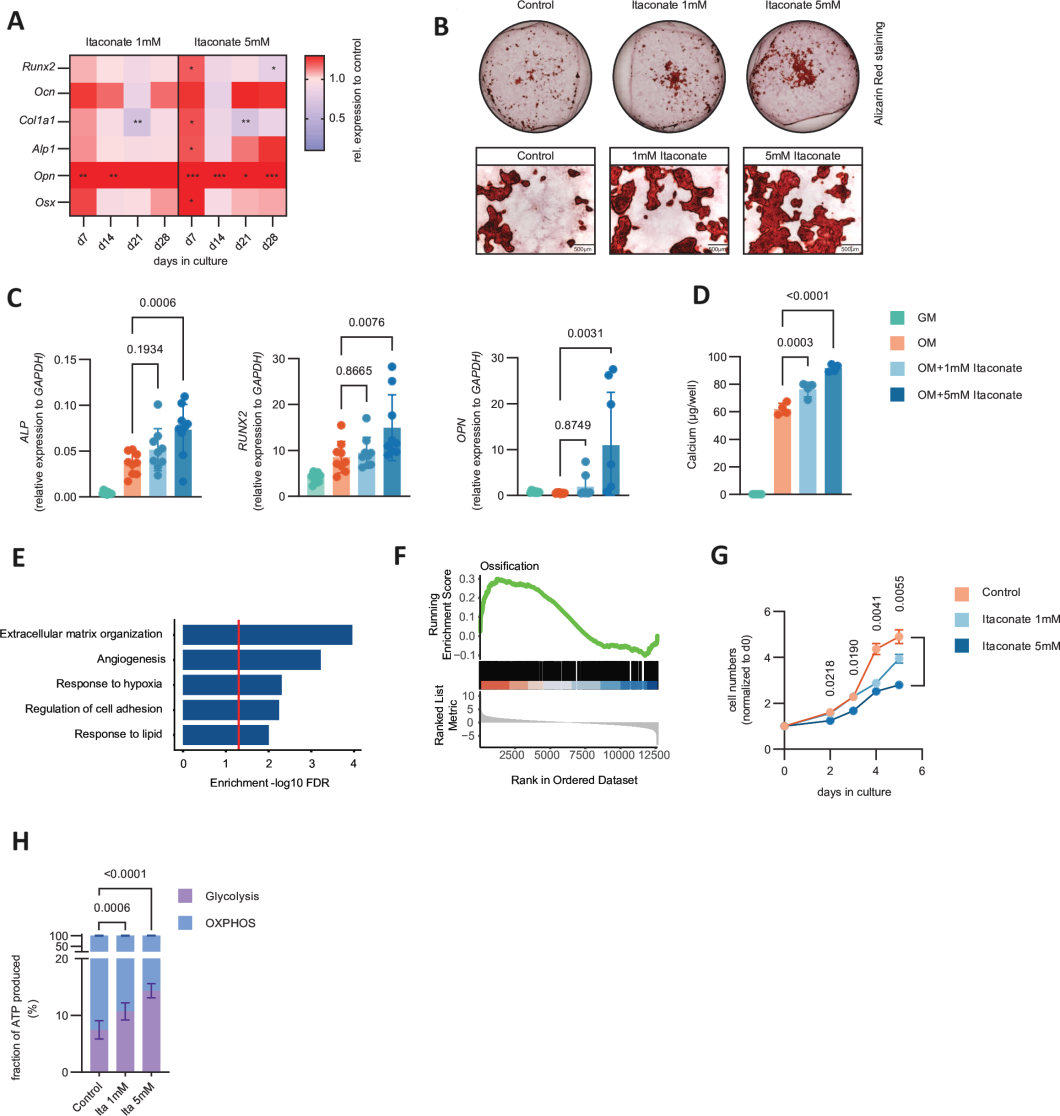
Itaconate stimulates osteoblast function in vitro. (A) Heatmap for relative expression of osteoblast marker genes at indicated time points during osteogenic differentiation of murine calvarial osteoblasts cultivated in medium supplemented with 1 mM or 5 mM itaconate at indicated time points determined by real-time PCR compared with control (n=4). (B) Representative pictures of Alizarin Red staining of calvarial osteoblasts in control osteogenic differentiation medium and medium supplemented with 1 mM or 5 mM itaconate. Top pictures show wells of 12-well plate and pictures below show snapshots (scale bar represents 500 µm). (C) Relative expression of osteoblast marker genes of human bone marrow stromal cells cultivated in growth medium (GM), osteogenic differentiation medium (OM) and OM supplemented with 1 mM or 5 mM itaconate using real-time PCR (n=9). (D) Calcium deposits in wells from human bone marrow stromal cells cultivated as shown in (C) (n=4). (E) Gene ontology enrichment (FDR<0.1) of differentially regulated genes in human bone marrow stromal cells cultivated in OM versus OM supplemented with 5 mM itaconate. (F) Gene set enrichment analysis for GO:0001503 (ossification, 333 genes) of differentially regulated genes in human bone marrow stromal cells cultivated in OM versus OM supplemented with 5 mM itaconate. (G) Cell number of murine calvarial osteoblasts normalised to day 0 in control growth medium or supplemented with either 1 mM or 5 mM itaconate (n=4). (H) Fraction of total ATP produced by glycolysis or oxidative phosphorylation (OXPHOS) of MC3T3-E1 cells in control medium or medium supplemented with either 1 mM or 5 mM itaconate. The data are represented as means±SDs. Unpaired t-test (A), one-way ANOVA with Šídák’s correction (C, D), two-way ANOVA with Tukey’s correction (G) and mixed-effect analysis with Šídák’s correction (H). Asterisks in (D) indicate p values: *<0.05, **<0.01, **<0.001. ANOVA, analysis of variance.

Based on these results, we aimed to explore if itaconate affects osteoblast function. Itaconate was readily taken up by osteoblasts and in line with its described inhibitory effects on succinate dehydrogenase dose dependently increased intracellular succinate levels (online supplemental figure S5a, b).^16^ Importantly, we observed that itaconate increased expression of both transcription factors implicated in osteogenic differentiation and genes encoding for extracellular matrix proteins associated with bone formation (figure 5A). Addition of succinate mimicked the stimulatory effect of itaconate on some osteoblast markers (*Runx2*, a trend also for *Col1a1* and *Ocn*), but not others (*Alp*, *Opn*, *Osx*) (online supplemental figure S5c), suggesting that succinate contributes to the effect of itaconate on osteoblasts. In line, coculturing osteoblasts with RANKL-stimulated BMCs from *Irg1^+/+^* animals resulted in higher expression of the osteoblast markers *Runx2* and *Col1a1* compared with coculture with RANKL-stimulated BMCs from *Irg1^−/−^* animals (online supplemental figure S5d). We also detected increased bone nodule formation in osteoblast cultures supplemented with itaconate (figure 5B and online supplemental figure S5e). The stimulatory effect of itaconate on osteoblast-associated gene expression was similar in human osteoblast cultures as itaconate upregulated genes like *ALP*, *RUNX2*, *BSP*, *OPN* and *COL1A1* (figure 5C and online supplemental figure S5f), which also resulted in higher calcium deposition and alkaline phosphatase (ALP) activity (figure 5D and online supplemental figure S5g, h). RNA-seq of osteoblasts showed that globally only a small number of genes were differentially regulated on itaconate stimulation compared with controls (online supplemental figure S5i). GO term enrichment analysis revealed that extracellular matrix reorganisation was among the most affected processes and GSEA showed enrichment of ossification-related genes in itaconate-treated cells (figure 5E, F and online supplemental table 3). As preosteoblasts start to enter the differentiation phase, it has been shown that they stop proliferating and subsequently increase their glycolytic activity.^27^,^29^ When we added itaconate during the proliferation phase of osteoblast cultures, we detected a dose-dependent inhibition of proliferation (figure 5G and online supplemental figure S5j). In agreement with the notion that itaconate stimulates osteoblast differentiation, it also enhanced the glycolytic activity of osteoblasts relative to oxidative phosphorylation (figure 5H). We also assessed a potential cell autonomous effect of *Irg1* in osteoblasts by determining the mRNA and protein levels of this gene in proliferating osteoblasts or during osteogenic differentiation and could not detect any expression of *Irg1* (online supplemental figure S6a, b). Moreover, we could also not observe clear differences of osteoblast marker genes between osteoblasts isolated from *Irg1*^−/−^ and *Irg1*^+/+^ mice (online supplemental figure S6c).

**Figure 6:**
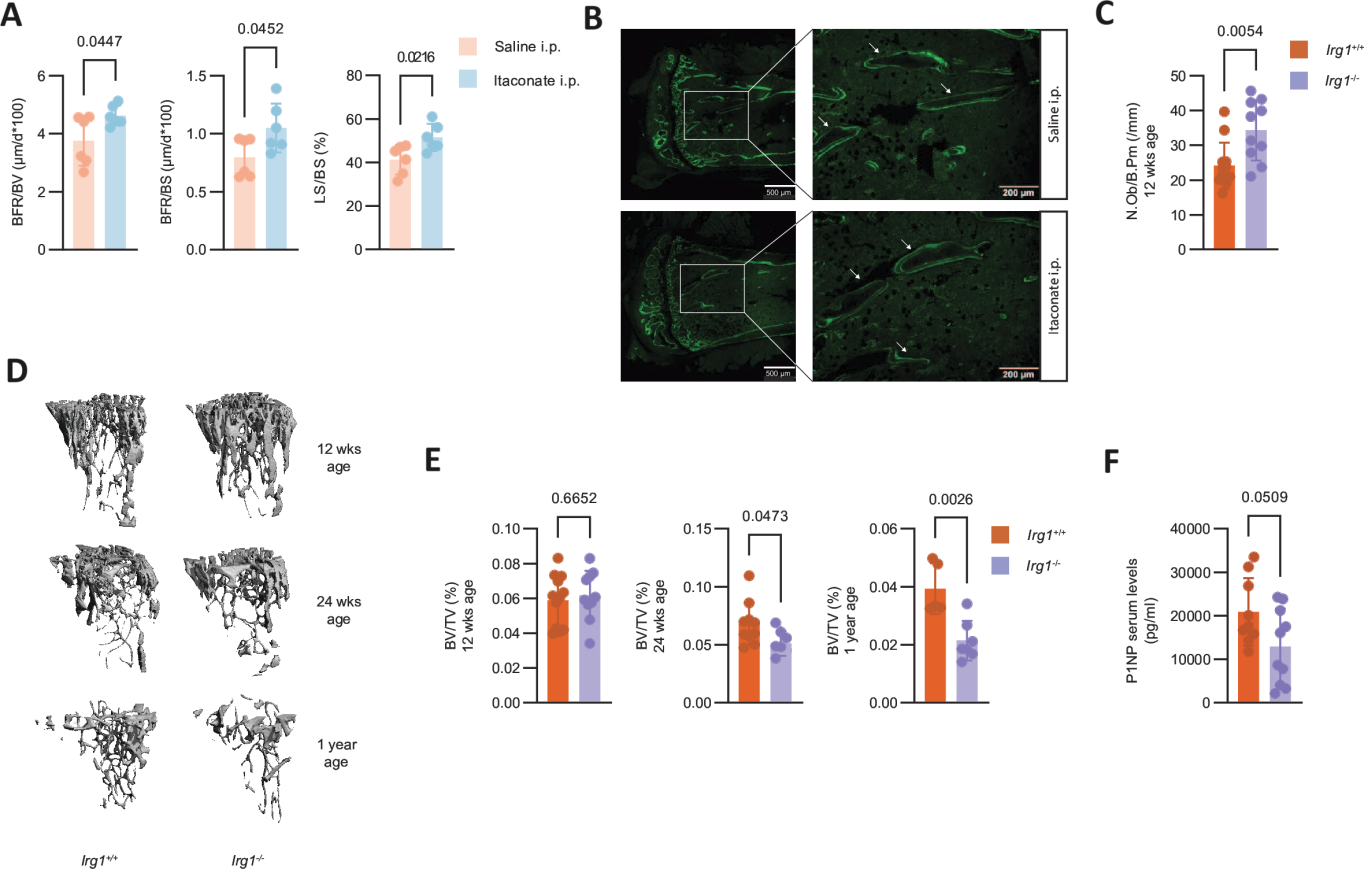
Itaconate stimulates osteoblast function in vivo. (A) Bone formation rate per bone volume (BFR/BV (µm/d×100)), bone formation rate per bone surface (BFR/BS (µm/d×100)) and labelled surface per bone surface (LS/BS) of tibial bones from mice daily receiving intraperitoneal injections with either saline or itaconate as assessed by calcein labelling (n=6). (B) Representative fluorescence images of calcein labelled bone from mice shown in (A). White arrows indicate surfaces with fluorescent mono layers or double layers. Scale bars represents 500 µm and 200 µm. (C) Number of osteoblasts per bone perimeter (No.OB/B.PM (/mm)) of tibial bones from 12-weeks-old *Irg1^+/+^ and Irg1^−/−^* mice (n=10–12). (D) Representative 3D reconstructions of tibiae from 12 weeks, 24 weeks and 1-year-old *Irg1^−/−-^* or *Irg1^+/+^* mice. (E) Bone volume per tissue volume (BV/TV (%)) of tibiae from 12 weeks, 24 weeks and 1-year-old *Irg1^−/−^* or *Irg1^+/+^* mice assessed by µCT analysis (n=5–12). (F) Serum levels of N-Terminal Propeptide Of Type I Procollagen (P1NP) from 24 weeks old *Irg1^+/+^* and *Irg1^−/−^* mice as determined with ELISA (n=10). The data are represented as means±SDs. Unpaired t-test (A, C, E, F). *Irg1*, immunoresponsive gene 1.

Having established the effects of itaconate on osteoblast in vitro, we tested if itaconate also affects osteoblasts in vivo and used calcein to label new bone formation in mice which were intraperitoneally injected with itaconate or saline for 7 days.^30^ Itaconate injections resulted in a twofold increase of itaconate above physiological levels in bone and a threefold increase in serum (online supplemental figure S7a, b). Notably, after 7 days of daily injections, we detected increased calcein labelling in mice that received itaconate compared with saline, resulting in increased double-labelled surface/bone surface (dLS/BS), double-labelled surface/labelled surface (dLS/LS), mineralised surface/bone surface (MS/BS), but no difference in mineral apposition rate, suggesting that itaconate stimulates osteoblast maturation also in vivo, without altering the activity of individual osteoblasts (figure 6A, B and online supplemental figure S7c). Hypothesising that itaconate also regulates osteoblast function or numbers to maintain bone homeostasis, we analysed bones of *Irg1*^+/+^ and *Irg1*^−/−^ mice. We found that the N.Ob/B.Pm and Ob.S/BS were increased in 12 weeks old *Irg1*^−/−^ mice compared with *Irg1*^+/+^ mice and in 15-weeks-old mice of the ovariectomy experiment, consistent with an inhibitory effect of endogenous itaconate on osteoblast proliferation in vivo (figure 6C and online supplemental figure S8a, b). However, this effect was no longer detectable after 24 weeks (online supplemental figure S8c). While bone mass was not different in 12-weeks-old mice, we detected a decreased bone volume to tissue volume ratio in 24 weeks and 1-year old and a reduction of procollagen type 1 N-Terminal peptide (P1NP) concentrations in serum by about 40% in 24-weeks-old *Irg1*^−/−^ compared with *Irg1*^+/+^ mice (figure 6D–F).

## Discussion

Together, our data reveal that osteoclastogenesis is associated with a profound metabolic rewiring distinctly marked by the accumulation of intracellular and extracellular itaconate. We further demonstrate that this metabolite serves as a stimulator of osteogenic differentiation. Since its discovery in activated macrophages in 2011, itaconate emerged as a prime example for an autocrine immunoregulatory metabolite, most notably inhibiting inflammasome activation and increasing type I interferon responses.^1831^,^35^ Although itaconate secretion has been reported already in earlier studies,^16 18^ it has only recently been shown that environmental itaconate mediates paracrine effects on stromal cells in specific settings like lung fibrosis and non-alcoholic fatty liver disease.^36^,^38^ In our analysis of differentiating osteoclasts, itaconate was the most abundant extracellular TCA cycle intermediate. We also detected other TCA cycle metabolites including succinate, which has been implicated as a paracrine regulator of bone homeostasis by affecting osteoclast generation in hyperglycaemic mice.^39^ However, in contrast to itaconate, none of the other TCA cycle metabolites measured accumulated over time during osteoclastogenesis, suggesting that itaconate is not metabolised further, rendering it a suitable paracrine mediator. The vicinity of osteoclasts and osteoblasts possibly allows for high local concentrations of itaconate that lead to the observed effects on osteoblasts including decreased proliferation and boosted differentiation and function. The stimulatory effect of itaconate was consistent in murine and human osteoblasts. Our findings resemble the effects of other osteoanabolic stimuli such as parathyroid hormone or Wnt proteins, which have been demonstrated to increase glycolysis in osteoblastic cells.^28 40 41^

The role of itaconate and its widely used derivatives such as four-octyl itaconate (4-OI) as potential drugs has been evaluated in a variety of conditions including osteoclast generation, where addition of 4-OI has been demonstrated to inhibit osteoclast formation in vitro and in vivo.^31 42 43^ The authors imply activation of Nuclear factor erythroid 2-related factor 2 (NRF2) to mediate this effect. Importantly, recent findings have highlighted significant differences between unmodified and modified itaconate as the relative electrophilicity of 4-OI and dimethylitaconate is higher than unmodified itaconate and can induce a stronger electrophilic stress response with manifold consequences including a potent activation of the NRF2 pathway response.^32^ We conclude that itaconate derivatives should be cautiously evaluated when studying the role of itaconate metabolism in a physiological context.

Interestingly, we also find that *Irg1-*deficient mice do not develop severe osteoproliferative lesions in the K/BxN serum transfer arthritis model. In line, we could observe that *Irg1* expressing cells cluster around the insertion sites of tendons into the bone, which is the preferred location of new bone formation.^24^ These lesions are also found in patients suffering from psoriatic arthritis or ankylosing spondylitis and predominantly develop at joint locations with strong infiltration of inflammatory cells which have high osteoclastogenic potential.^24^ Itaconate generated by these cells might therefore play a role in endochondral new bone formation, which significantly contributes to the pathology and symptom burden of these diseases by causing functional impairment and pain. While myeloid cells expressing IRG1 in tendovaginitis are likely also macrophages and/or neutrophil granulocytes and not only osteoclast precursors, we propose that in arthritis also other myeloid cells produce itaconate to stimulate osteoblasts. However, colocalisation of TRAP and *Irg1* expressing cells next to the tendon insertion sites support the concept that cells ultimately differentiating into osteoclasts participate in driving osteoproliferative lesions.

In conclusion, our data reveal a previously unrecognised role of itaconate in the fine tuning between myeloid cells such as osteoclasts and bone-forming osteoblasts. The links between itaconate, osteoblast differentiation and the development of osteoproliferative lesions reported in our study suggest *Irg1* and its related metabolites as potential drug targets for diseases which affect the bone and joints.

## Materials and methods

### Animals and disease models

Mice were bred and housed in a specific pathogen-free facility at the Medical University of Vienna and kept in a 12 hours light cycle at 21°C–23°C. Wildtype animals (C57BL/6J, RRID: IMSR_JAX:000664) for BMCs extraction and in vivo arthritis models were purchased from Charles River Laboratories or used from in-house breedings. K/BxN serum transfer arthritis was induced by intraperitoneal injection of 100 µL serum of K/BxN mice on day 0 and day 2 in C57BL/6J mice or *Irg1^−/−^* and *Irg1^+/+^* mice (C57BL/6NJ-Acod1em1(IMPC)J/J, RRID:IMSR_JAX:029340) from The Jackson Laboratory. Animals were harvested 12 days after the first injection of serum.^44^ *Irg1^−/−^* and *Irg1^+/+^* mice were kept in the same mouse facility and under the same conditions and identified by PCR using the following primers: 5′-GTGGGGAGGGGAACTATGAG-3′ (common), 5′-ATTTGGAGGAACCCCATGAC-3′ (WT) and 5′-CAGCCTCTAAGCCAGACAGC-3′ (Mutant). Human tumour necrosis factor (h*TNF*Tg/+, RRID: MGI:3053718) transgenic animals were identified by PCR using h*TNF*Tg primers (5′-TACCCCCTCCTTCAGACACC-3′ and 5′-GCCCTTCATAATATCCCCCA-3′) and were harvested at 8 weeks of age. h*TNF*Tg/+ mice were a kind gift of George Kollias^20^ and crossed with *Irg1^−/−^* to create h*TNF*Tg/+/*Irg1^−/−^* and h*TNF*Tg/+/*Irg1^+/+^* mice. CD11c-DTR mice (B6.FVB-1700016L21Rik^Tg(Itgax-HBEGF/EGFP)57Lan^/J, RRID:IMSR_JAX:004509) and B6 CD45.1 mice (B6.SJL-Ptprc^a^ Pepc^b^/BoyJ, RRID:IMSR_JAX:002014) were bought from The Jackson Laboratory. Ovariectomy of 10-weeks-old female *Irg1^−/−^* and *Irg1^+/+^* animals was performed as described previously.^45^ Briefly, ovaries were removed for ovariectomy while skin and peritoneum of sham-operated female animals was incised, and ovaries were left intact. After 4 weeks of recovery mice were sacrificed. Bone loss was evaluated in the tibiae using μCT image analysis. For itaconate injections, mice were intraperitoneally injected with 400 µL saline or pH-adjusted itaconate (Sigma, #I29204) solution (50 mg/mL) for 7 days on a daily basis and harvested at day 8. For RANKL injections, mice were intravenously injected with 50 µL PBS or RANKL (Abcam #ab129136) dissolved in PBS (0.4 µg/µL). For depletion of CD11c cells in CD11c-DTR mice during K/BxN serum transfer arthritis, 200 µL of diphtheria toxin dissolved in PBS (150 ng/mL) was intraperitoneally injected every 3 days starting on the day of arthritis induction. For depletion of CD11c cells in CD11c-DTR mice during homeostasis, mice were intraperitoneally injected once with 200 µL diphtheria toxin dissolved in PBS (150 ng/mL). All animal procedures were approved by the local ethics committee of the Medical University Vienna and were conducted in accordance with Austrian law.

### Clinical assessment of murine arthritis

Clinical signs of arthritis were assessed by a routinely used semiquantitative double-blind score system.^46^ Swelling per paw was recorded: 0–3 (0=no swelling; 1=mild swelling of toes/ankle; 2=moderate swelling of toes/ankle; 3=severe swelling of toes/ankle). Grip strength per paw was assessed on a wire mesh: 0–3 (0=normal grip strength; 1=mildly reduced grip strength; 2=moderately reduced grip strength; 3=severely reduced grip strength). Total score was calculated by combining scores of swelling and grip strength of all four paws.

### Histological analyses

Histological assessment was performed as previously described.^7^ Briefly, hind paws were fixed in 7.5% buffered formalin solution for 6 hours and then decalcified in 14% pH 7.2 EDTA/ammonium hydroxide buffer (Sigma, #ED-1kg, #318604) at 4°C for at least 7 days. Two micrometre decalcified paraffin-embedded sections were prepared and stained with H&E and TRAP (Sigma, #387A, Leukocyte Phosphatase Staining Kit). After deparaffinisation and rehydration, slides were stained 10 min with 1:5 diluted Meyer’s hemalum (Merck, #1.09249.0500), rinsed with distilled water, differentiated in 1% HCl ethanol and rinsed 10 min in tap water. Afterwards, slides were stained in eosin working solution (300 mL 1% Eosin, Sigma, #318906, 600 mL distilled water, 0.1 mL acetic acid 100%) for 15 s. Slides were then rinsed with distilled water, 96% ethanol, 100% ethanol, n-Butyl-acetate and mounted with Neo-Mount (Merck, #1.09016). For TRAP staining, slides were preincubated 1 hour at 37°C with the TRAP staining solution (250 µL naphthol AS-BI phosphoric acid, 1000 µL acetate solution, 500 µL tartrate solution, 45 mL distilled water; Sigma, #387A) protected from light. Afterwards, slides were developed for 2 min at 37°C with a mix of 250 µL of Fast garnet GBC base solution and 250 µL of sodium nitrite solution. Nuclei were stained with Meyer’s hemalum as described above (without HCl) and slides mounted in Aquatex (Merck, #108562). H&E and tartrate-resistant acid phosphatase stained sections were analysed using an Axioskop 2 microscope (Carl Zeiss MicroImaging) and Osteomeasure Analysis System (OsteoMetrics) to quantify the areas of joint inflammation, erosion, osteoclast numbers and osteoproliferative lesions according to the SMASH recommendations.^47^

### RNA in situ hybridisation

For RNA in situ hybridisation, bones and paws were fixed in 7.5% buffered formalin solution at 4°C overnight and then decalcified in 14% pH 7.2 EDTA/ammonium hydroxide buffer (Sigma, #ED-1kg, #318604) at 4°C for at least 7 days. Two micrometre decalcified paraffin-embedded sections were deparaffinised by incubating the slides at 60°C for 1 hour and rinsed three times with Neo-clear solution (Sigma # 1098435000) for 7 min. Slides were moved into 100% EtOH and afterwards air-dried at room temperature. After drying, RNAscope Hydrogen Peroxide (#322300) was added on the slides and incubated for 10 min at room temperature. Slides were washed with ddH_2_O for three times and then transferred into a staining dish filled with 95°C ddH_2_O for 10 s. Then the slides were transferred into a staining dish filled with 1× RNAscope target retrieval solution (#322000) and incubated at 95°C for 15 min. After target retrieval, slides were washed with ddH_2_O and dehydrated with 100% ethanol for 3 min. Slides were then air dried at room temperature. After creating a hydrophobic barrier two drops of RNAscope Protease Plus (#322300) were added and incubated for 30 min at 40°C in the HybEZ oven (#321720). Slides were then washed three times with ddH_2_O. Afterwards slides were incubated with the RNAscope probes Mm-Irg1-Mus musculus immunoresponsive gene 1 (*Irg1*) (#450241) or Negative Control (*dapB*) (#310043) and incubated for 2 hours at 40°C in the HybEZ oven. Slides were then washed two times with 1× RNAscope washing buffer (#310091). Slides were then incubated with the RNAscope HD detection reagents AMP 1–6 (#322310) for 30 min at 40°C (AMP1 and 3) or 15 min at 40°C (AMP2 and 4) or 30 min at room temperature (AMP5) or 15 min at room temperature (AMP6) with two washing steps with 1× RNAscope washing buffer between each AMP reagent solution. After the last washing step slides were incubated with RNAscope DAB solution (equal volumes of DAB-A and DAB-B) and incubated for 10 min. Then slides were washed with ddH_2_O and stained with Meyer’s hemalum (Merck, #1.09249.0500) as described above.

### Analysis of bone parameters

Histomorphometry and bone formation analysis of mouse tibia was analysed as described previously.^45^ For µCT analysis the SCANCO Medical µCT 35 was used to produce images from trabecular bone and analysed with SCANCO evaluation software for segmentation, three-dimensional morphometric analysis, density and distance parameters.

### Dynamic labelling of bone

Calcein labelling was performed and analysed as previously described.^46^ In short, mice were subcutaneously injected with calcein green (Sigma, #C0875-5G) (30 mg/kg) at day 7 and day 1 before harvest. Left tibial bones were embedded in methoxymethylmetacrylate. Measurements were performed on the entire marrow region within the cortical shell using OsteoMeasure.

### Bone marrow transplantations

Bone marrow from *Irg1*^−/−^, *Irg1*^+/+^ and B6 CD45.1 animals was isolated by flushing out cells from the femur and tibia with sterile PBS. Lethally irradiated (9 Gy) 8–12-week-old *Irg1*^−/−^, *Irg1*^+/+^ and B6 CD45.1-recipient mice were injected retro-orbitally with 3×10^6^ cells under anaesthesia and were allowed to recover for 8 weeks. Chimerism was confirmed by flow cytometry of the blood. After recovery, arthritis was induced by intraperitoneal injection of 150 µL serum of K/BxN mice on day 0 and day 2.

### Osteoclastogenesis and staining

Osteoclast assays were performed as previously described.^7^ In brief, bone marrow cells were isolated and non-adhering haematopoietic stem cells were cultured in MEMα (Gibco, #32561037) completed with 1% Pen-Strep (Gibco, #15140122) and 10% fetal calf serum (FCS, Gibco, #10082147) supplemented with 100 ng/mL M-CSF (R&D Systems, #416). After 3 days, cells were washed with PBS, scraped, replated and cultured in complete MEMα supplemented with 30 ng/mL M-CSF and/or 50 ng/mL RANKL (R&D Systems, #462) for another 3–4 days including one medium change on day 6. Osteoclasts were defined as TRAP positive (TRAP staining kit, Sigma, #387A) multinucleated cells (≥3 nuclei). For glutamine-free and glucose-free media, MEMα powder without nucleosides, glucose, pyruvate, vitamin C, amino acids (Genaxxon, #C4002.0010) was dissolved in ddH_2_O and supplemented with all amino acids used in the formulation of Gibco MEMa except from glutamine. Dialysed FCS (Gibco, #26400036) was used for the assays with glutamine-free and glucose-free medium. For osteoclast assays showing the influence of α-KG addition to glutamine-free medium, dimethyl 2-oxolglutarate (Sigma, #349631) was used. For RANKL stimulation of HoxB8 precursor cells,^17^ HoxB8 cells were cultured in MEMα (Gibco, #32561037) completed with 1% Pen-Strep (Gibco, #15140122) and 10% FCS (Gibco, #10082147) supplemented with 100 ng/mL M-CSF (R&D Systems, #416). After 3 days, cells were washed with PBS, and medium was changed with complete MEMα supplemented with 30 ng/mL M-CSF and/or 50 ng/mL RANKL (R&D Systems, #462). For RANKL stimulation of sorted human CD14^+^ monocytes, classical monocytes (CD14^+^ CD16^−^) were sorted from peripheral blood mononuclear cells of healthy donors after density gradient centrifugation and cultured in MEMα (Gibco, #32561037) completed with 1% Pen-Strep (Gibco, #15140122) and 10% FCS (Gibco, #10082147) supplemented with human 50 ng/mL M-CSF (R&D systems, #216-MC). After 3 days, medium was changed with complete MEMα supplemented with 30 ng/mL human M-CSF and/or 50 ng/mL human RANKL (R&D Systems, #6449-TEC). Human tissue collection was conducted in accordance with the Medical University of Vienna Review Board ID 1075/2021.

### Osteoblast cultures

Osteoblast cultures of murine cells were performed as previously described.^45^ In brief, osteogenic precursor cells were isolated from calvariae of neonatal mice. Calvariae were digested for 10 min per fraction and a total of five fractions in MEMα (Gibco, #32561037) containing 0.1% Collagenase (Sigma, #10103586001) and 0.2% Dispase II were collected (Sigma, #D4693). Osteogenic precursors were expanded in MEMα containing 10% FCS (Gibco) and 1% Pen-Strep (Gibco, #15140122). Differentiation of osteoblasts was achieved using mineralisation medium by adding 0.2 mM l-ascorbate (Sigma, #A5960) and 5 mM β-glycerophosphate (Sigma, #G9422). Medium was replaced every 2–3 days. Itaconate (Sigma, #I29204) was dissolved in 1M NaOH solution, pH-adjusted to 7.4 and added at a final concentration of 1 mM or 5 mM. After 26 days, cells were fixed with 7.5% buffered formalin solution, following staining with ddH_2_O containing 8% Alizarin Red (Sigma, #A5533). Cell number was assessed by detaching cells with trypsin and counting them with a TC20 automated cell counter (Bio-Rad). For diethylsuccinate (Sigma #112402) treatment of osteoblasts, mineralisation medium was supplemented with 100 µM diethylsuccinate. For coculture experiments, osteogenic precursors were isolated and expanded as described above. Bone marrow cells from *Irg1^+/+^* and *Irg1^−/−^* mice were isolated as described above and cultured in MEMα completed with 1% Pen-Strep and 10% FCS supplemented with 100 ng/mL M-CSF. After 3 days, cells were washed with PBS, scraped and seeded onto the osteogenic precursors. Differentiation of osteoblasts was achieved using mineralisation medium by adding 0.2 mM l-ascorbate and 5 mM β-glycerophosphate and osteoclastic differentiation was induced by addition of 30 ng/mL M-CSF and/or 50 ng/mL RANKL.

For the human osteoblast cultures, commercially available human bone marrow mononuclear cells (Lonza, #LON2M-125C) were expanded in standard growth medium (DMEM high glucose (HG) supplemented with 10% FCS, 1% L-glutamine, 1% Pen-Strep and 1 ng/mL basic fibroblast growth factor (Fisher Scientific #100-18B)). Aliquots were frozen in cryopreservation medium (standard growth medium containing 10% DMSO) in liquid nitrogen. After cultivation, cells were switched to osteogenic differentiation medium (DMEM HG containing 10% FCS, 1% L-glutamine, 1% Pen-Strep, 10 nM dexamethasone, 50 µM ascorbic acid-2-phosphate, 10 mM ß-glycerophosphate) (Sigma #A8960, #G9422, #D4902) supplemented with or without 1 mM or 5 mM itaconate. Medium change was performed twice per week. After 3 weeks, samples for ALP enzyme activity and mature osteoblast marker genes were harvested. After one additional week, samples for Alizarin Red S (Sigma #A5533) staining as well as for calcium deposition determination were harvested.

### Real-time PCR

Total RNA was extracted from cells using TRIzol reagent (Invitrogen, #12034977) and Monarch RNA Clean-up kit (NEB, #T2030L). Reverse-transcription was performed using High-Capacity cDNA Reverse Transcription Kit (Applied Biosystems, #4368814). Luna Universal qPCR Master Mix (NEB, #M3003E) was used for the quantitative PCR reaction. For data with murine cells to obtain sample-specific ΔCt values, normalisation to hypoxanthine phosphoribosyltransferase 1 (*Hprt*) (for data with murine BMCs, murine calvarial osteoblasts, HoxB8 cells, cross-organ *Irg1* comparison), glyceraldehyde 3-phosphate dehydrogenase (*GAPDH*) (for data with tibial bone and paws) and beta-actin (*Actb*) (for data with diethylsuccinate treatment) within each sample was performed. Data are shown as fold change, where 2−ΔΔCt values were calculated (ΔΔCt=ΔCt treatment−ΔCt control). For data with human cells, normalisation to *GAPDH* (for human bone marrow stromal cells) or to beta-actin (*ACTB*) (for human sorted CD14^+^ monocytes) within each sample was performed. Real-time PCR was performed using the following primers for murine genes: *Hprt*: 5′-CGCAGTCCCAGCGTCGTG-3′ and 5′-CCATCTCCTTCATGACATC TCGAG-3′; *Actinb*: 5'-TGTCCACCTTCCAGCAGATGT-3' and 5'-AGCTCAGTAACAGTCCGCCTAGA-3', *Gapdh*: 5'TCGTCCCGTAGACAAAATGG-3' and 5'TTGAGGTCAATGAAGGGGTC-'3; *Nfatc1*: 5′-GACAGACATCGGGAGGAAGA-3′ and 5′-AGCCTTCT CCACGAAAATGA-3′; *Jdp2*: 5′-CTCCTCCTGCTATGATGCCT-3′ and 5′-CTCTTGCCCAGTTTCACCTC-3′; *Ocstamp*: 5′-ATGAGGACCATCAGGGCAGCCACG-3′ and 5′-GGAGAAGCTGGGTCAGTAGTTCGT-3′; *Dcstamp*: 5′-TCCTCCATGAACAAACAGTTCCAA-3′ and 5′-AGACGTGGTTTAGGAATGCAGCTC-3′; *Fos*: 5′-AGCCCAGACCT GCAGTGGCT-3′ and 5′-GCGCTCTGCCTCCTGACACG-3′, *Irg1*: 5′-GCGAACGCTGCCACTCA-3′ and 5′-ATCCCAGGCTTGGAAGGTC-3′, *Runx2*: 5′-CGGAGCGGACGAGGCAAGAGTTTC-3′ and 5′-AGACAGCGGCGTGGTGGAGTGGAT-3′, *Ocn*: 5′-ACCCTGGCTGCGCTCTGTCTCT-3′, *Col1a1*: 5′-CTGACTGGAAGAGCGGAGAG-3′ and 5′-GCACAGACGGCTGAGTAGG-3′, *Alp1*: 5′-GCTGATCATTCCCACGTTTT-3′ and 5′-CTGGGCCTGGTAGTTGTTGT-3′, *Opn*: 5′-CTCCTTGCGCCACAGAATG-3′ and 5′-TGGGCAACAGGGATGACA-3′, *Osx*: 5′-AGCGACCACTTGAGCAAACAT-3′ and 5′-GCGGCTGATTGGCTTCTTCT-3′, *Itgam*: 5′-CATCAAGGGCAGCCAGATTG-3' and 5'-GAGGCAAGGGACACACTGAC-3', *Emr1*: 5'-CTTTGGCTATGGGCTTCCAGTC-3' and 5'-GCAAGGAGGACAGAGTTTATCGTG-3'. Real-time PCR was performed using the following primers for human genes: *ACTB*: 5'-ATTGCCGACAGGATGCAGAA-3' and 5'- GCTGATCCACATCTGCTGGAA-3'; *IRG1*: 5'-ATGCTGCTTTTGTGAACGGTG-3' and 5'-CTACCACGGAAGGGGGATGGA-3'. For human bone marrow stromal cells TaqMan universal PCR master mix and the following TaqMan Gene Expression Assays (Thermo Fisher Scientific) were used: *GAPDH* (Hs02786624_g1), *ALP* (Hs01029144_m1), *OPN* (Hs00959010_m1), *BSP* (Hs00173720_m1), COL1A1 (Hs00164004_m1) and RunX2 (Hs04940094_m1).

### Western blot

Cells were washed with ice-cold PBS and lysed with RIPA lysis buffer system (Santa Cruz, #24948). The homogenate was centrifuged at 4°C for 15 min at 16 000 g and the supernatant containing the protein fraction recovered. Protein concentration in the supernatant was determined using the Pierce BCA Protein Assay Kit (Thermo Fisher Scientific, #23225). A total of 10 µg of proteins were resolved by SDS-PAGE and transferred to PVDF membranes (GE Healthcare, #10600023). Membranes were blocked with TBS-Tween (0.1%) containing 5% skim milk powder (Sigma, #70166) and incubated with constant agitation with primary antibody solution at 4°C overnight. The following antibodies were used: IRG1 (Abcam, #ab222411, 1:1000) and beta-ACTIN (Cell Signaling #4970, 1:2000). Incubated membranes were washed three times for 5 min with TBS-Tween (0.1%) and probed with a goat antirabbit horseradish peroxidase-linked secondary antibody (Cell Signaling, #7074, 1:10,000). Antigen-specific binding of antibodies was detected with WesternBright Sirius HRP substrate (Advansta, #541020). Western blots were assessed by an area density analysis using the Vision Works Software (Analytik Jena).

### RNA sequencing

For RNA-seq data of murine BMCs stimulated with RANKL, total RNA was extracted from cells using TRIzol reagent (Invitrogen, #12034977) and Monarch RNA Clean-up kit (NEB, #T2030L). NGS libraries were prepared using the QuantSeq 3′ mRNA-Seq FWD library preparation protocol (Lexogen GmbH, Vienna, Austria) and sequenced on a HiSeq 3000 instrument (Illumina, San Diego, California, USA) following a 50-base-pair, single-end recipe. HiSeq Control Software (HCS, HD V.3.4.0.38) and Real-Time Analysis Software (RTA, V.2.7.7) were used for raw data acquisition and base calling, respectively. Raw fastq files were quality and adapter trimmed using *Trim galore!* (V.0.4.4) followed by alignment to the mouse genome GRCm38 using STAR (V.2.5.2^48^). R software (V.4.0.4) and DESeq2 package (V.1.30.1) was used to calculate differential gene expression and a |shrunkFC|>1.5 and a FDR<0.05 were defined as cut-offs.^49^ Functional enrichment analyses were performed using the Curated.WikiPathways database implemented in ShinyGO (V.0.76^50^). Only pathways containing at least five genes were considered.

For RNA seq data of human bone marrow stromal cells, RNA was extracted using TRIzol reagent and a standard protocol for acid-guanidium-phenol-chloroform extraction with RNA precipitation by isopropanol and washing with ethanol. RNA pellet was resuspended in nuclease-free water and stored at −80°C until further use. Libraries were prepared with the QuantSeq 3′ mRNA-Seq V2 Library Prep Kit with UDI (Lexogen, Austria) according to the manufacturer’s instructions. A 250 ng total RNA was used as input and final amplification of libraries was performed with 17 PCR cycles. Libraries were pooled in equimolar ratio and sequenced on Illumina NovaSeq SP Flowcell in SR100 mode. Overall quality of the next-generation sequencing data was evaluated automatically and manually with fastQC V.0.11.8 and multiQC V.1.7. Reads from all passing samples were adapter trimmed and quality filtered using bbduk from the bbmap package V.38.69 and filtered for a minimum length of 17nt and phred quality of 30. Alignment steps were performed with STAR V.2.7 using samtools V.1.9 for indexing, whereas reads were mapped against the genomic reference GRCh38.p12 provided by Ensembl. Assignment of features to the mapped reads was done with htseq-count v0.13. The human gene-level read counts were further analysed in R V.4.2.1. The read counts were normalised by total number of all mappable reads (library size) for each gene. The limma voom (V.3.52.4) results in a matrix of normalised gene expression values on log2 scale. The counts and normalised log2 limma voom expression values were used as a raw input for all the analysis. Outlier samples were checked by principal component analysis. The genes that showed expression below one count per million (cpm<1) in the group of replicates were excluded from downstream analysis. To calculate differential expression for human data we used |logFC|>0.5 and FDR<0.1. Functional enrichment analysis were performed using ShinyGO V.0.77 and GSEA using fgsea R package (v1.25.1).

### Data availability and code availability

Raw RNA-seq data were deposited at the Gene Expression Omnibus (GEO) database (GSE208359, for mouse data) and (GSE236452, for human data). The data code is available from the corresponding authors on reasonable request.

### U-^13^C glucose and U-^13^C glutamine isotope tracing

Cells were cultured for the indicated time points in the presence of fully labelled glucose (Cambridge Isotope Laboratories, #CLM-1396–1) and/or fully labelled glutamine (Cambridge Isotope Laboratories, CLM-1822-H-0.1). Metabolites were extracted using methanol:water (80:20, v/v) and extracts were centrifuged for 10 min at 5000 g. The supernatant was collected and dried using a nitrogen evaporator. The samples were reconstituted in 50 µL of methanol, centrifuged for 10 min at 1000 g and supernatant was used for LC-MS analysis. For AA tracing, a Vanquish UHPLC system (Thermo Scientific) coupled to an Orbitrap Fusion Lumos (Thermo Scientific) mass spectrometer was used for the LC–MS analysis. The chromatographic separation was carried out on an ACQUITY UPLC BEH Amide, 1.7 µm, 2.1×100 mm analytical column (Waters) equipped with a VanGuard: BEH Amide, 2.1×5 mm pre-column (Waters). The column was maintained at a temperature of 40°C and 2 µL sample were injected per run. The mobile phase A was 0.15% formic acid (v/v) in water with 10 mM ammonium formate and mobile phase B was 0.15% formic acid (v/v) in 85% ACN (v/v) with 10 mM ammonium formate. The flow rate was 0.4 mL/min and gradient elution was performed with a total analysis time of 17 min. The mass spectrometer was operated in a positive electrospray ionisation mode: spray voltage 3.5kV; sheath gas flow rate 60arb; auxiliary gas flow rate 20arb; capillary temperature 285°C. For the analysis a full MS scan mode with a scan range *m/z* 50–250, resolution 500 000; AGC target 2e5 and a maximum injection time 50 ms was applied. The data processing was performed with the TraceFinder 4.1 software (Thermo Scientific). For TCA cycle tracing, a Vanquish UHPLC system (Thermo Scientific) coupled to an Orbitrap Fusion Lumos (Thermo Scientific) mass spectrometer was used for the LC-MS analysis. The chromatographic separation was carried out on an ACQUITY HSS T3, 1.8 µm, 2.1×100 mm analytical column (Waters) equipped with a VanGuard HSS T3, 2.1×5 mm pre-column (Waters). The column was maintained at a temperature of 40°C and 2 µL of sample was injected per run. The mobile phase A was 0.1% formic acid (v/v) in water and mobile phase B was 0.1% formic acid (v/v) in methanol. The flow rate was 0.5 mL/min and gradient elution was performed with a total analysis time of 10 min. The mass spectrometer was operated both in positive and negative electrospray ionisation mode: spray voltage was 3.5kV for positive mode and 3.0kV for negative mode; sheath gas flow rate 60arb; auxillary gas flow rate 20arb; capillary temperature 285°C. For the analysis a full MS scan mode with a scan range *m/z* 80–400, resolution 500 000; AGC target 2e5 and a maximum injection time 50 ms was applied. The data processing was performed with the TraceFinder 4.1 software (Thermo Scientific).

### Metabolomics

For determining TCA cycle intermediates from *Irg1^+/+^* and *Irg1^−/−^* cells, metabolite extracts were generated by removing medium and washing once with cold PBS. After addition of −20°C quenching solution (methanol:acetonitrile:water, 2:2:1 v/v) plates were covered with dry ice for 15 min. After thawing on wet ice, cells were scraped and transferred to a microcentrifuge tube. After three cycles of freeze and thaw with liquid nitrogen, extracts were left on −20°C for 1 hour and spun down with 15 000 g for 15 min. Supernatant was transferred to a new tube and stored at −80°C. For determining itaconate levels from tibial bone or paws, tissue was snap frozen in liquid nitrogen and grinded with liquid nitrogen in a metal morter. Grinded tissue powder was transferred to microcentrifuge tubes and 500 µL of addition of −20°C quenching solution (methanol:acetonitrile:water, 2:2:1 v/v) was added. After three freeze and thaw cycles with liquid nitrogen, extracts were left on −20°C for 1 hour and spun down with 15 000 g for 15 min. For determining itaconate in serum samples, blood was collected in microcentrifuge tubes and stored at room temperature for 1 hour. Blood was then spun down with 5000 g for 10 min and serum was transferred to new microcentrifuge tubes. Proteins were removed by adding 400 µL of a methanol/ethanol mixture (4:1, v/v) to 100 µL of plasma in a microcentrifuge tube followed by vigorous vortex shaking for 5 min at room temperature and centrifugation at 4000 g for 10 min at 4°C. The supernatant was collected, transferred to another microcentrifuge tube, shock frozen with liquid nitrogen and stored at −80°C until analysis. Metabolite extracts were analysed by hydrophilic interaction chromatography (HILIC), directly coupled to tandem mass spectrometry (LC-MS/MS). In brief, 1 µL of the original sample was directly injected onto a polymeric iHILIC-(P) Classic HPLC column (HILICON, 100×2.1 mm; 5 µm) and the respective guard column, operated at a flow rate of 100 µL/min. The HPLC (Ultimate 3000 HPLC system; Dionex, Thermo Fisher Scientific) was directly coupled via electrospray ionisation to a TSQ Quantiva mass spectrometer (Thermo Fisher Scientific). A linear gradient (A: 95% acetonitrile 5%, 10 mM aqueous ammonium acetate; B: 5 mM aqueous ammonium bicarbonate, both supplemented with 0.1 µg/mL medronic acid) starting with 15% B and ramping up to 60% B in 9 min was used for separation. Detection and quantification have been done by LC-MS/MS, employing the selected reaction monitoring (SRM) mode of the instrument. Authentic standards were used for determining collision energies and optimal transitions of the SRM and for validating experimental retention times via standard addition. The following SRM transitions were used for quantitation in the negative ion mode: *m/z* 115 to *m/z* 71 (fumarate), *m/z* 117 to *m/z* 73 (succinate), *m/z* 129 to *m/z* 85 (itaconate), *m/z* 133 to *m/z* 115 (malate), *m/z* 145 to *m/z* 101 (α-KG), *m/z* 173 to *m/z* 85 (aconitate), *m/z* 191 to *m/z* 111 (citrate) and *m/z* 808 to *m/z* 408 (acetyl-CoA). Data interpretation was performed using TraceFinder (Thermo Fisher Scientific).

### Real-time cell metabolic analysis

Oxygen consumption rate and extracellular acidification rate were measured on a Seahorse XF HS mini Analyzer (Agilent) using the Seahorse XF Real-Time ATP Rate Assay Kit (Agilent, #1 03 591-100) according to the manufacturer’s instructions. In brief, 5000 MC3T3-E1 osteogenic precursor cells (ATCC, #CRL-2593) were seeded per well in a XF HS mini plate using MEMα containing 10% FCS (Gibco) and 1% Pen-Strep (Gibco #15140122). After 3 days when cells reached full confluency, medium was replaced with mineralisation medium by adding 0.2 mM l-ascorbate (Sigma # A5960) and 2 mM β-glycerophosphate (Sigma #G9422) to complete MEMα with or without 1 mM or 5 mM itaconate. On the next day, medium was changed to XF DMEM medium (Agilent, #1 03 575–100) supplemented with or without itaconate (1 mM or 5 mM) and plates were kept in a non-CO_2_ incubator for 45 min for equilibration before measurement. During the measurements, oligomycin (1.5 µM) and rotenone/antimycin A (500 nM) were subsequently injected. Raw data were analysed using Seahorse Analytics Online Software system, exported as a Microsoft Excel file and graphed in GraphPad Prism V.9 (GraphPad Software).

### ALP enzyme activity assay

To determine alkaline phosphatase (ALP) activity, the samples were lysed in a solution containing 0.5% Triton X-100 in 0.5M 2-amino-2-methyl-1-propanol buffer with 2 mM MgCl_2_ (with pH 10.3) for 1 hour at 4°C. The lysate was centrifuged for 5 min at 300 g and ALP activity of the supernatant was determined by incubation with 0.02M p-nitrophenyl phosphate substrate solution at 37°C. The reaction time until sufficient development of colour change was recorded and the reaction was stopped by adding 0.2M NaOH stop solution. Absorbance was measured at 405 nm, and the ALP activity was determined using a standard curve constructed with p-nitrophenol solutions of known concentrations.

### Calcium concentration assay

For calcium content quantification, samples were extracted using 5% trichloroacetic acid at RT for 30 min. The samples were collected, centrifuged for 10 min at 4°C and the calcium content of the supernatant was determined using the calcium (CPC) LiquiColor test (Stanbio Laboratory, Boerne, USA) according to manufacturer’s instructions.

### ELISA

ELISA-based measurements of CTX-1 and P1NP levels were performed according to the manufacture’s protocol using the Cross Linked C-Telopeptide Of Type I Collagen (CTXI) ELISA kit (Cloud-Cone Corp., #CEA665Mu) and the Mouse P1NP (Procollagen I N-Terminal Propeptide) ELISA Kit (Elabscience, # E-EL-M0233).

### Statistics

Statistical analysis was performed by using a two-tailed t-test, an ordinary one-way ANOVA followed by Tukey’s, Šidàk’s or Dunnet’s multiple comparison tests if comparing selected pairs of means or a two-way ANOVA followed by Tukey’s or Šidàk’s multiple comparisons test with Prism V.9 software (GraphPad, La Jolla, California). All error bars indicate±SD

## supplementary material

10.1136/ard-2023-224898 online supplemental file 1

10.1136/ard-2023-224898 online supplemental file 2

10.1136/ard-2023-224898 online supplemental file 3

10.1136/ard-2023-224898 online supplemental file 4

10.1136/ard-2023-224898 online supplemental file 5

10.1136/ard-2023-224898 online supplemental file 6

10.1136/ard-2023-224898 online supplemental file 7

10.1136/ard-2023-224898 online supplemental file 8

10.1136/ard-2023-224898 online supplemental file 9

10.1136/ard-2023-224898 online supplemental file 10

10.1136/ard-2023-224898 online supplemental file 11

## Data Availability

Data are available in a public, open access repository. Data are available upon reasonable request.
