# Supplementary figures and images for: Itaconate is a metabolic regulator of bone formation in homeostasis and arthritis
Source: Ann Rheum Dis. 2024 Jul 10;83(11):e224898. doi: 10.1136/ard-2023-224898 (PMC11503170; doi:10.1136/ard-2023-224898)

# Figure S1

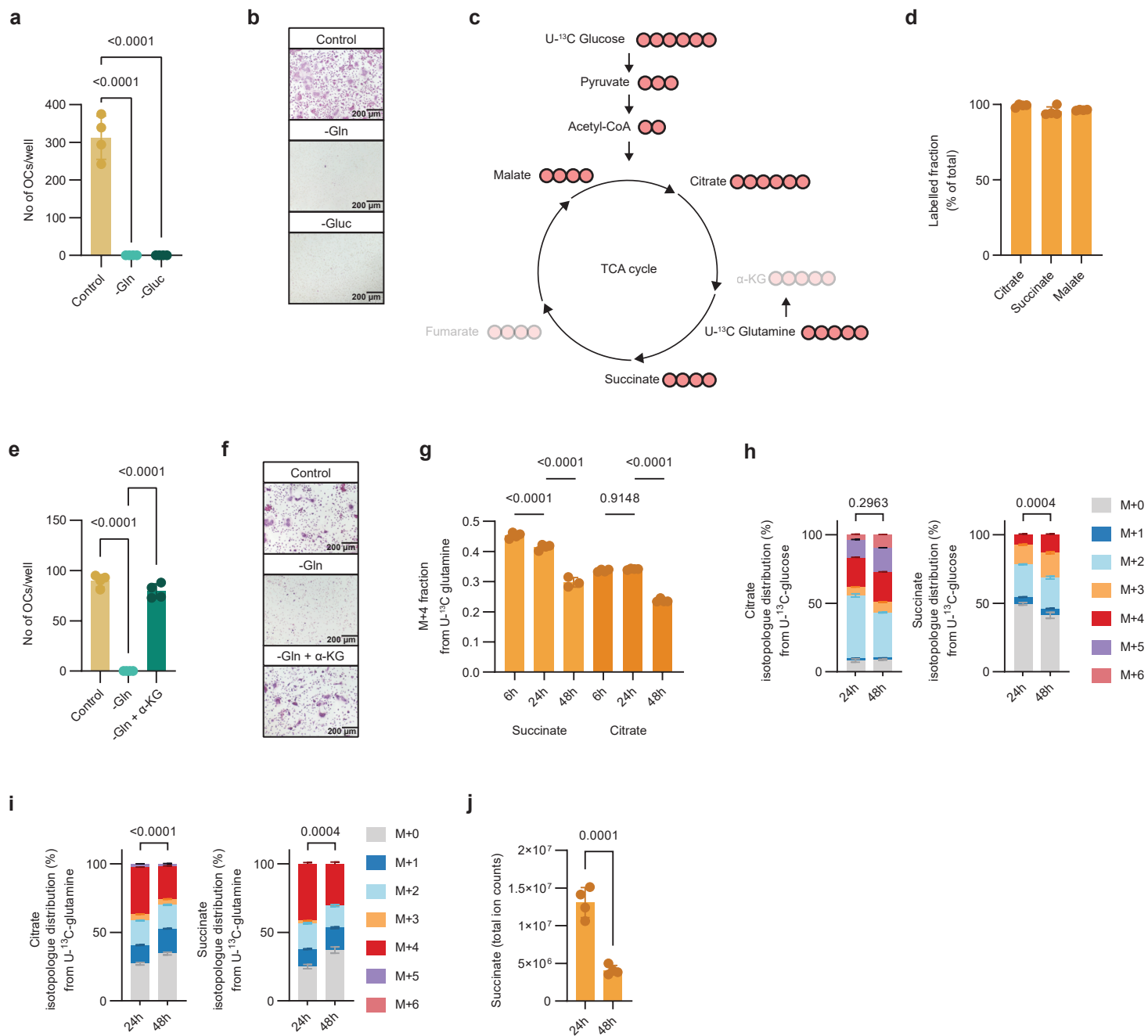

Supplement: online supplemental file 1 [file ard-83-11-s001.pdf]

Figure S2

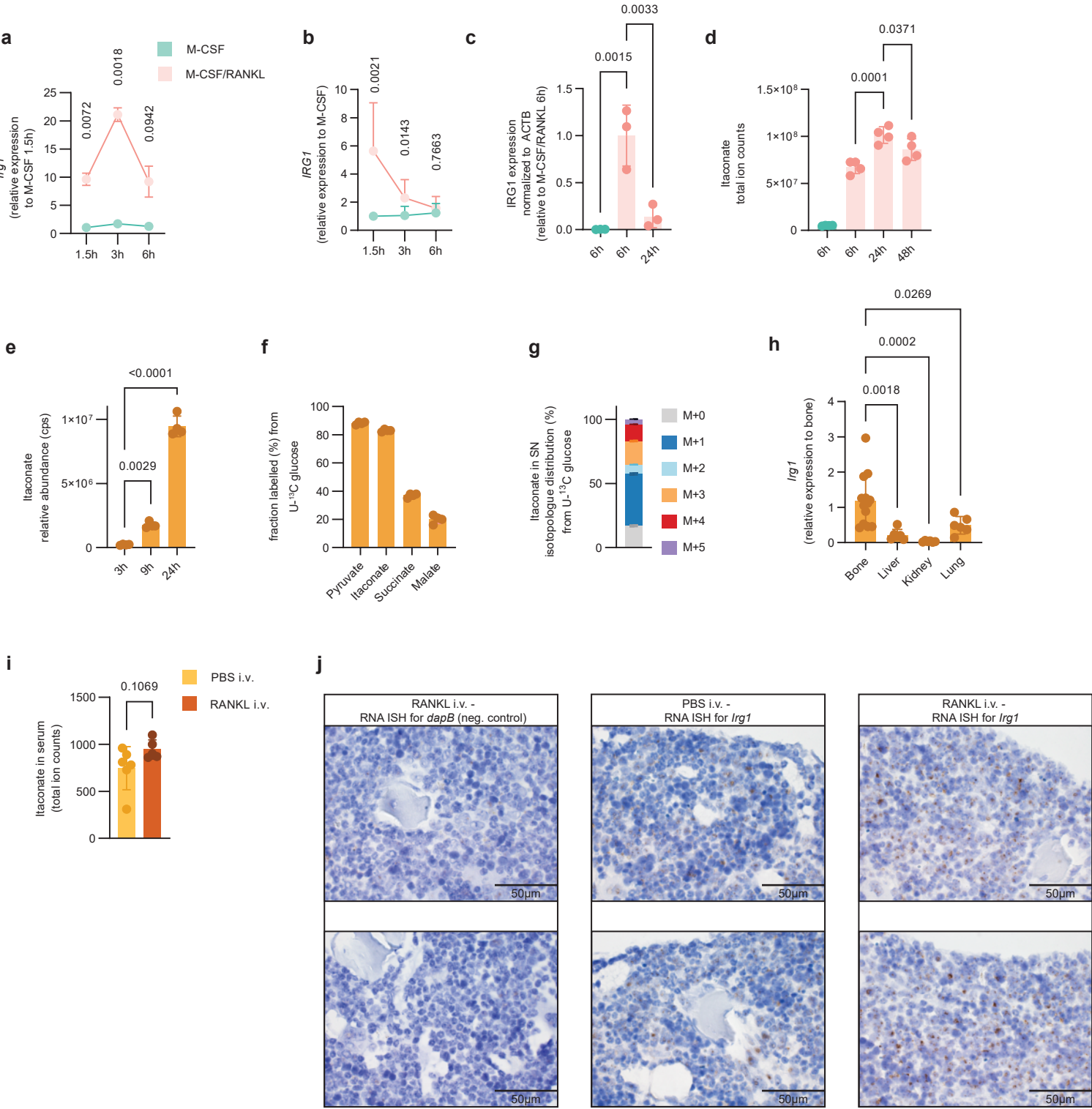

Supplement: online supplemental file 2 [file ard-83-11-s002.pdf]

Figure S3

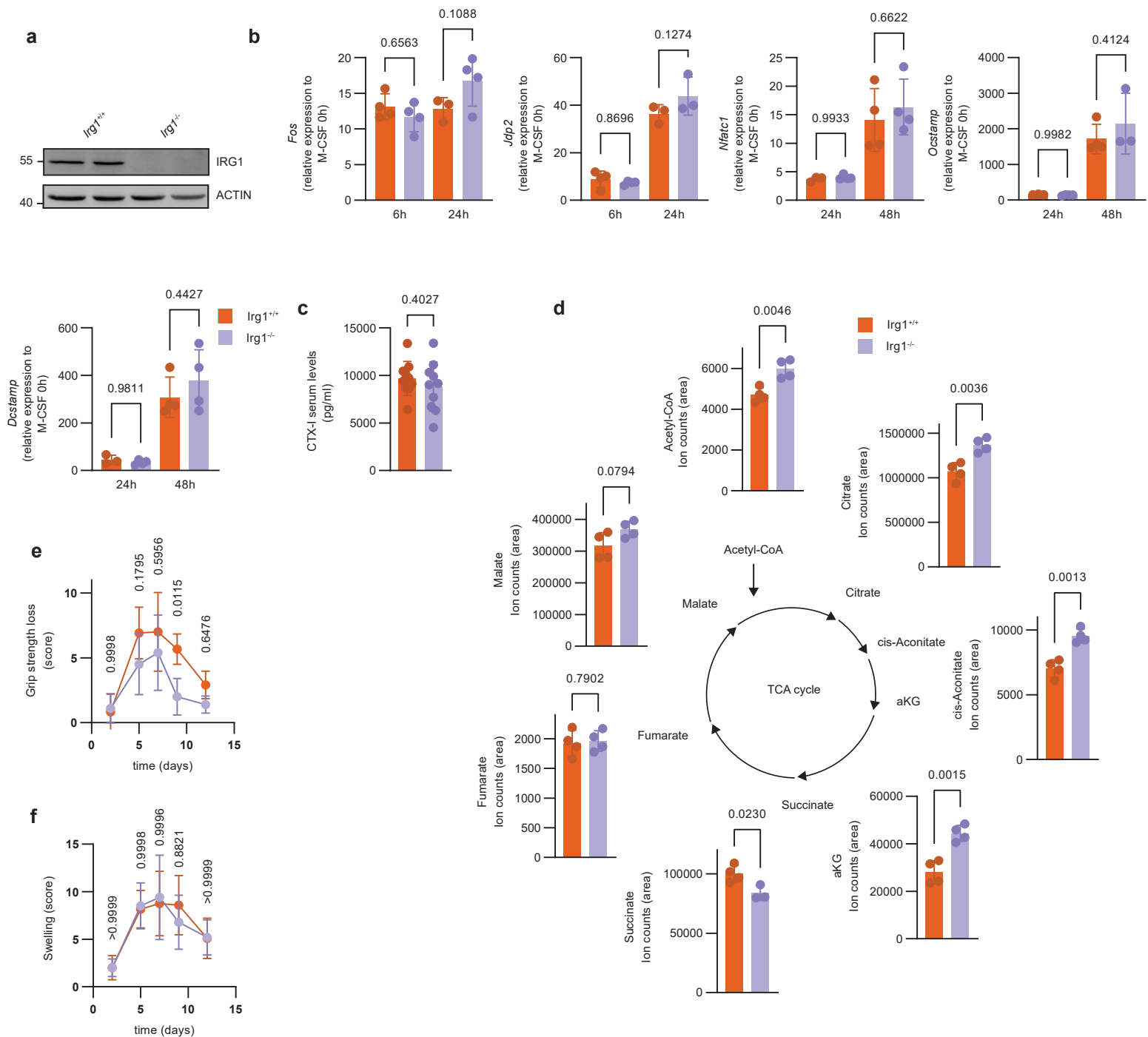

Supplement: online supplemental file 3 [file ard-83-11-s003.pdf]

Figure S4

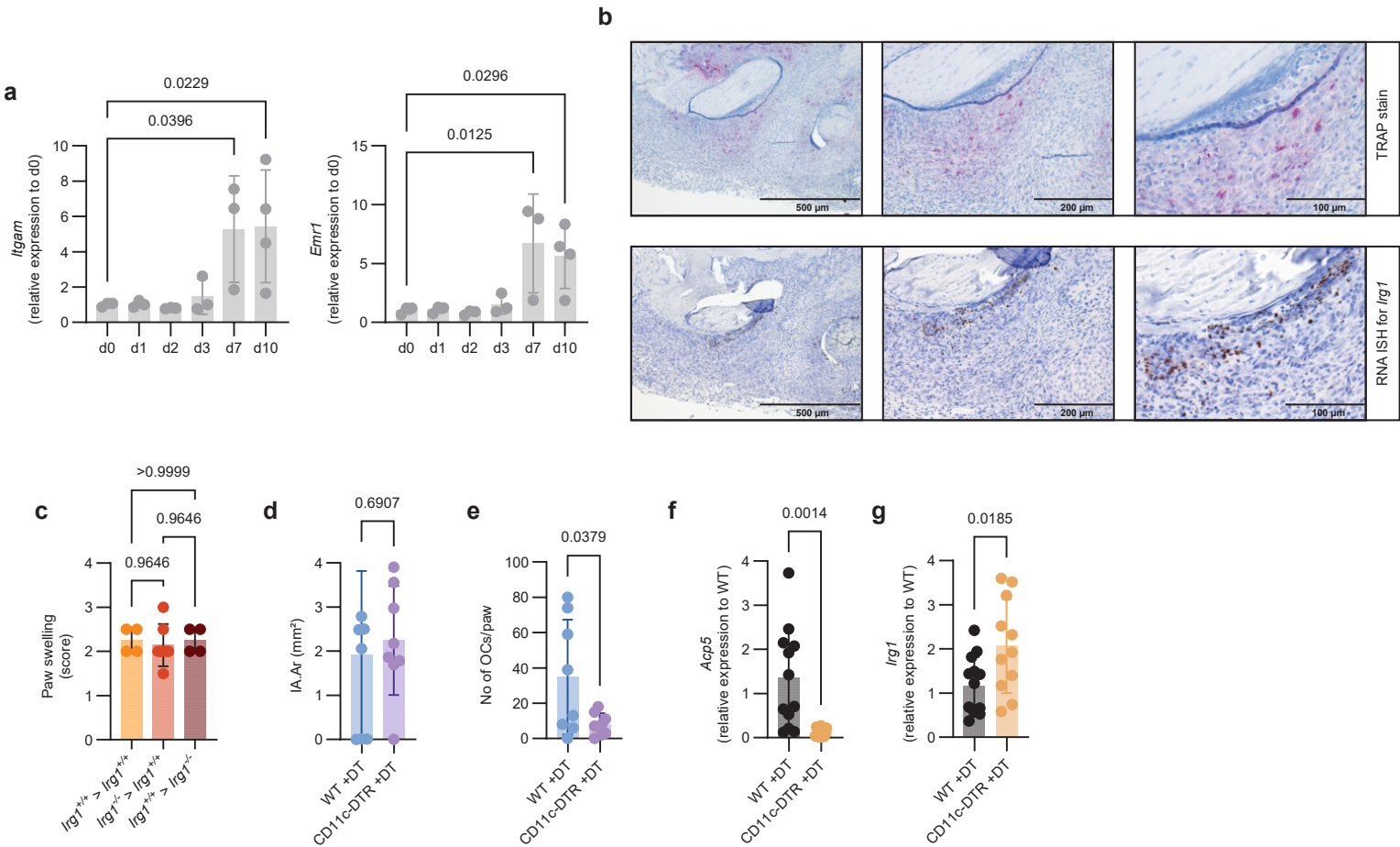

Supplement: online supplemental file 4 [file ard-83-11-s004.pdf]

Figure S5

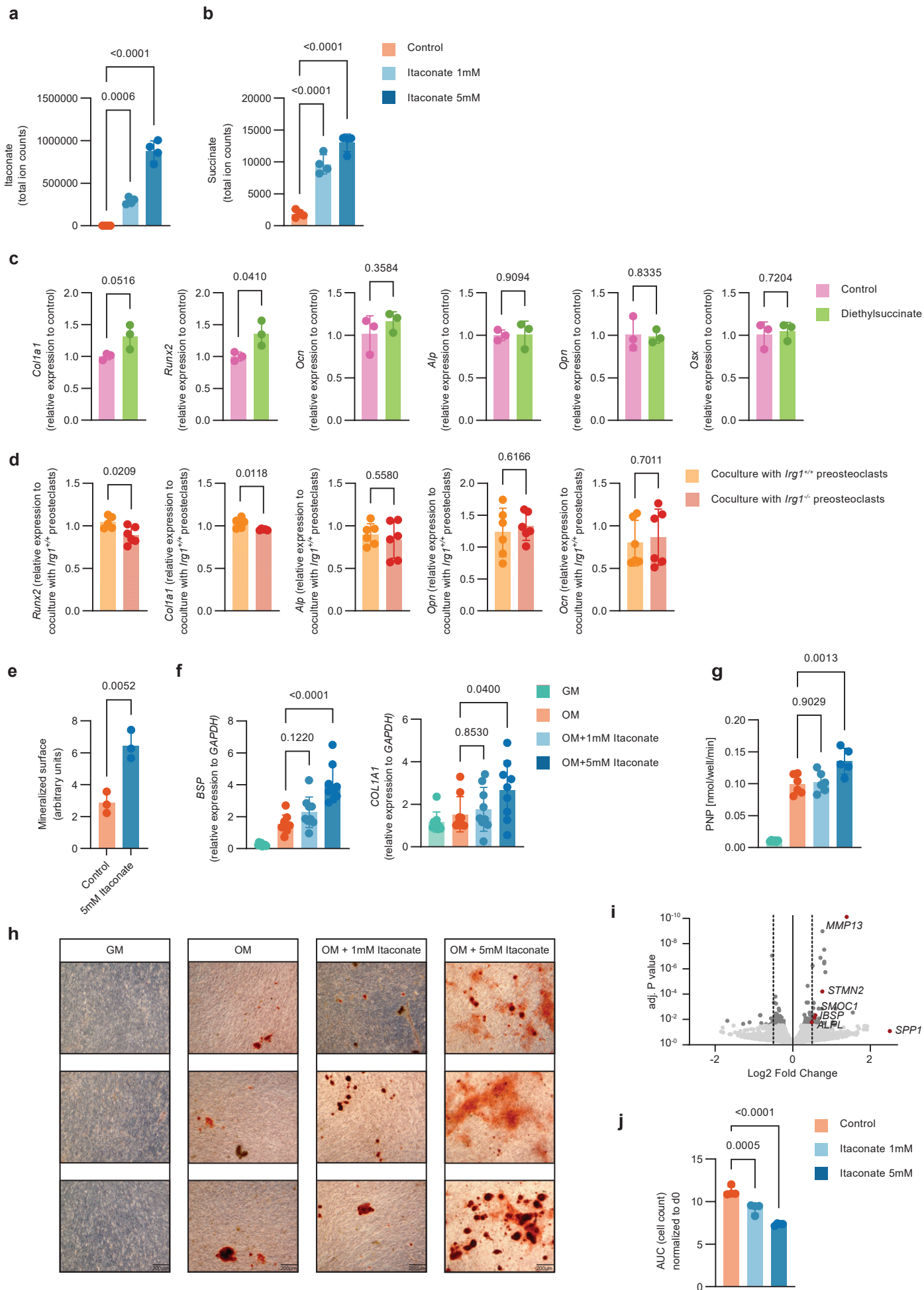

Supplement: online supplemental file 5 [file ard-83-11-s005.pdf]

Figure S6

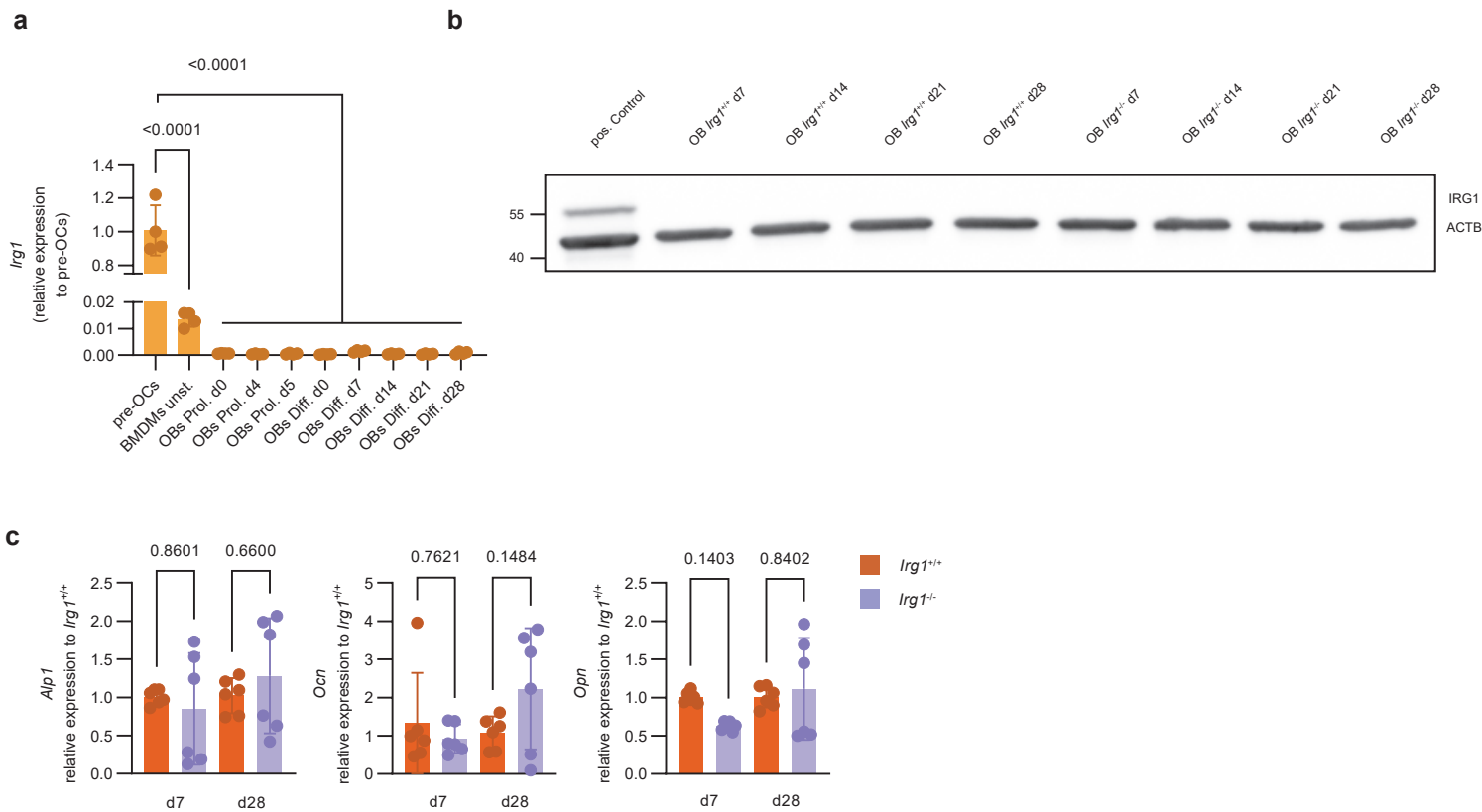

Supplement: online supplemental file 6 [file ard-83-11-s006.pdf]

Figure S7

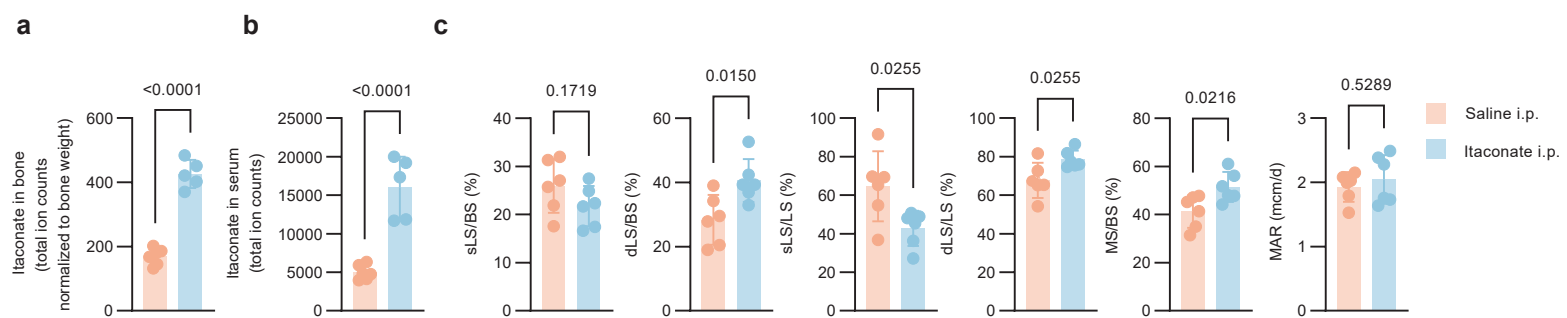

Supplement: online supplemental file 7 [file ard-83-11-s007.pdf]

Figure S8

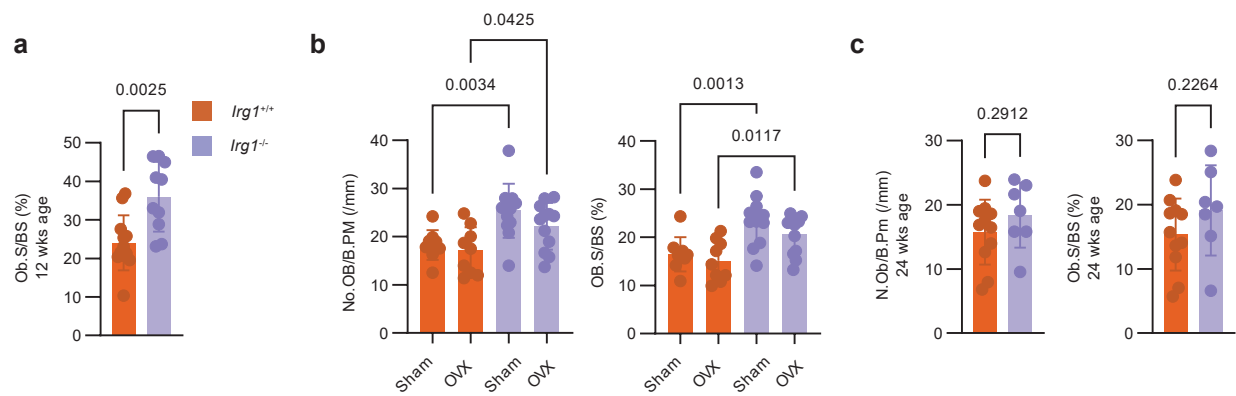

Supplement: online supplemental file 8 [file ard-83-11-s008.pdf]
